# Post-Acute Care Pathways After Sexual Violence and Intimate Partner Violence: An International Health-Services Scoping Review with Implications for Italy

**DOI:** 10.3390/healthcare14121735

**Published:** 2026-06-16

**Authors:** Paolo Bailo, Chiara Carsana, Maria Garreffa, Anna Carannante, Marco Giustini, Cecilia Fazio, Loredana Falzano, Iris Locatelli, Valentina Strappa, Maria Simonetta Spada, Matteo Marchesi, Andrea Piccinini, Simona Gaudi

**Affiliations:** 1 Section of Legal Medicine, School of Law, University of Camerino, 62032 Camerino, Italy; paolo.bailo@unicam.it; 2 Section of Legal Medicine and Insurance Medicine, Department of Biomedical Sciences for Health, University of Milan, 20133 Milan, Italy; chiara.carsana1@unimi.it (C.C.); maria.garreffa@unimi.it (M.G.); 3 Department of Environment and Health, Istituto Superiore di Sanità, 00161 Rome, Italy; anna.carannante@iss.it (A.C.); marco.giustini@iss.it (M.G.); cecilia.fazio@iss.it (C.F.); simona.gaudi@iss.it (S.G.); 4 National Center for Global Health, Istituto Superiore di Sanità, 00161 Rome, Italy; loredana.falzano@iss.it; 5 School of Specialization in Health Psychology, University of Bergamo, 24129 Bergamo, Italy; i.locatellli@studenti.unibg.it; 6 Clinical Psychology Unit, ASST Papa Giovanni XXIII, 24127 Bergamo, Italy; vstrappa@asst-pg23.it (V.S.); sspada@asst-pg23.it (M.S.S.); 7 Medicolegal Unit, Hospital Division, ASST Papa Giovanni XXIII, 24127 Bergamo, Italy; matteo.marchesi@unimib.it

**Keywords:** scoping review, post-acute care, care pathways, sexual violence, intimate partner violence, continuity of care, trauma-informed care, advocacy, case management, health services

## Abstract

**Highlights:**

**What are the main findings?**

- Post-acute care pathways remain fragmented and inconsistently defined across sexual violence and IPV services.
- More continuity-oriented models combine structured follow-up, coordinated navigation, and multi-component support over time.

**What are the implications of the main findings?**

- The findings support evaluating strategies to reduce passive referral and strengthen phased follow-up pathways supported by warm handoffs and care coordination.
- Hybrid in-person and digital continuity models may support retention, access, and survivor-centred recovery when embedded within safe, trauma-informed services.

**Abstract:**

**Background/Objectives:** Survivors of sexual violence and domestic violence/intimate partner violence (IPV) often require support beyond the immediate emergency encounter; however, post-acute care remains inconsistently defined, unevenly organised or conceptualised, and fragmented across service systems. This scoping review mapped international post-acute follow-up, care, assistance, and support pathways, with particular attention to organisational models, continuity mechanisms, loss to follow-up after first access, and implications for the Italian context. **Methods:** We conducted an international health-services scoping review of post-acute follow-up, care, assistance, and support interventions for survivors of sexual violence and domestic violence/IPV. Searches were performed in PubMed/MEDLINE, Scopus, Web of Science Core Collection, Embase, APA PsycINFO via EBSCOhost, and CINAHL via EBSCOhost. Eligible studies were published from 2013 onward and had to describe an identifiable post-acute component beyond the initial emergency, forensic, or first-contact phase. The review followed a Population–Concept–Context framework and was reported in accordance with PRISMA-ScR. **Results:** Forty-four studies were included in the core synthesis, comprising 16 studies on sexual violence/sexual assault, 27 on domestic violence/IPV, and one mixed domestic, family, and sexual violence outreach model. The sexual violence literature clustered around early trauma-focused interventions, sexual assault care centre pathways, medical follow-up, follow-up attendance, and digital continuity tools. The IPV literature was broader and included psychotherapy, advocacy and case-management models, housing-first and trauma-informed stabilisation approaches, nurse-led and clinic-based services, outreach and safety-contact programmes, digital interventions, and programmes for system-involved survivors. Across both fields, the pathways most consistently described as supporting continuity combined structured re-contact, coordinated support, and multi-component responses over time. **Conclusions:** The mapped literature supports conceptualising post-acute responses to sexual violence and domestic violence/IPV as continuity pathways that extend beyond first contact and link healthcare, psychological, advocacy, and social supports. Systems may be better positioned to support continuity when they provide structured follow-up, warm handoffs, coordinated navigation, and context-sensitive recovery models. These findings point to provisional, evidence-informed organisational questions for strengthening post-acute pathways, including in Italy, particularly around structured re-contact, warm handoffs, survivor navigation, and integration between healthcare, anti-violence, psychological, and territorial social-support services.

## 1. Introduction

Violence against women, including sexual violence and domestic violence/intimate partner violence (IPV), remains a major public health, social, and human rights concern worldwide. Beyond the acute event, these forms of violence are associated with enduring physical, reproductive, psychological, and social consequences, while recovery trajectories are shaped by structural, economic, and sociocultural conditions rather than by clinical care alone [1,2,3]. This broader framing is important because the international literature increasingly suggests that post-acute responses should be understood not only as a matter of trauma treatment, but also as a question of continuity, equity, and survivor-defined recovery [4,5].

In recent years, increasing attention has been devoted to immediate response pathways, including emergency care, first-line support, forensic assessment, and early referral. By contrast, what happens after the first contact remains less clearly standardised and more weakly conceptualised. Major international guidance now distinguishes immediate first-line response from additional clinical care, mental health support, and broader continuity functions, while also emphasizing health-system strengthening and organisational readiness [2,6,7]. Empirical work in English Sexual Assault Referral Centres similarly suggests that mental-health assessment and onward referral can remain uneven even in dedicated sexual-assault services [8]. Emerging frameworks therefore move from generic trauma-informed care toward trauma-and-violence-informed care (TVIC), which explicitly incorporates structural violence, stigma, and intersecting inequities into service design [3,9].

This distinction is particularly important because post-acute pathways may determine whether an initial disclosure or healthcare encounter becomes the start of a coherent recovery process or merely an isolated event. The newer literature suggests that strong pathways should not be evaluated only through conventional service indicators such as attendance or referral uptake, but also through survivor-defined outcomes including empowerment, safety, autonomy, social connection, and broader experiences of healing [4,5]. In parallel, both scholarly and organisational sources emphasize that integrated models are often weakened by power imbalances within multidisciplinary teams, inconsistent communication, administrative burdens, and insufficient trauma-informed organisational culture [7,10,11].

The comparative dimension is especially relevant for the Italian context. Rather than focusing on legal doctrine alone, the present review is concerned with what is concretely offered after the acute phase: psychological follow-up, advocacy, coordination, stabilisation, and continuity across services. This question is particularly salient in Italy, where institutional guidance and national planning documents coexist with persistent underreporting, uneven regional implementation of integrated models such as Codice Rosa/Pink Code, and limited evaluation of long-term survivor outcomes and continuity pathways [12,13,14,15,16]. The Italian discussion should therefore move beyond the mere presence of services and ask whether survivors encounter a recognisable continuity pathway after first access, including psychological, social, and practical support over time.

A major unresolved gap in the field is the limited availability of international, post-acute, pathway-oriented syntheses—understood here as syntheses addressing the organisational sequence, coordinating functions, service-integration mechanisms, and outcome architecture of care after first contact—that move beyond acute and medico-legal responses to compare how continuity of care is organised, measured, and experienced by survivors across service systems [3,4,5,17]. Unlike reviews centred primarily on acute sexual assault response, forensic-medical intake, IPV screening, trauma-focused treatment, or single-service advocacy models, this review focuses on the organisational continuity mechanisms that operate after first contact and across healthcare, psychological, advocacy, community, and social-support sectors.

Accordingly, this review maps international evidence on post-acute follow-up, care, assistance, and support interventions offered to survivors of sexual violence and domestic violence/IPV after the initial emergency or first-contact phase, and discusses their potential implications for the Italian context. The review focuses on continuity mechanisms, service integration, survivor-centred engagement, and implementation-relevant features, rather than on acute response or medico-legal intake alone [3,4].

## 2. Methods

This study was conducted as an international scoping review of post-acute follow-up, care, assistance, and support interventions for survivors of sexual violence and domestic violence/intimate partner violence (IPV). A scoping review design was selected because the available evidence was conceptually, organisationally, and methodologically heterogeneous, and because the primary aim was to map and compare existing pathway models rather than to estimate pooled effectiveness for a single intervention or service model.

The review questions addressed the nature, organisation, and outcomes of post-acute pathways. The primary review question was: What concrete post-acute follow-up, care, assistance, and support interventions are offered to survivors of sexual violence and domestic violence/IPV in different countries after the initial emergency or first-contact phase? Secondary questions concerned the most frequent pathway components, the professionals and services involved, the settings and continuity mechanisms used, the outcomes reported, and the organisational implications that may be relevant for the Italian context.

No external protocol was registered, and no registration number is available. However, an internal review plan was defined before full screening and specified the review questions, Population–Concept–Context framework, eligibility criteria, database architecture, screening rules, and data-charting variables. Operational refinements during pilot testing were limited to calibration of the post-acute boundary and harmonisation of category assignment.

Eligibility criteria followed a Population–Concept–Context framework. The population included adolescent and adult survivors/victims of sexual violence, sexual assault, rape, domestic violence, and IPV. The concept was post-acute intervention, defined as any organised activity delivered after the initial emergency response, first forensic/medical assessment, or first service contact, with the aim of ensuring continuity of care, psychological recovery, social support, safety planning, clinical monitoring, advocacy, case management, or longer-term multidisciplinary follow-up. The context included any country and any healthcare, social care, community, or hybrid setting, including hospitals, sexual assault care/referral services, outpatient clinics, shelters, mental health services, anti-violence services, NGOs, territorial services, and integrated multidisciplinary networks.

Eligible interventions included scheduled clinical follow-up, psychological or psychiatric follow-up, trauma-focused psychotherapy, advocacy, case management, safety planning after the crisis phase, coordinated multidisciplinary care, community-based support, social reintegration services, telehealth follow-up, and other continuity-of-care models. For inclusion, the post-acute component had to be identifiable as more than immediate response alone and had to involve a recognisable continuity mechanism, follow-up structure, or recovery-oriented intervention beyond the first emergency, forensic, or first-access phase. For example, a scheduled medical or psychological review after emergency/forensic care, a safety-contact programme after an initial service encounter, a structured outreach pathway after disclosure, or an advocacy/case-management intervention maintaining contact beyond first access were considered eligible. Conversely, studies limited to acute forensic examination, emergency management, screening alone, hotline contact, initial disclosure, or service access without an identifiable continuity mechanism were excluded. Only peer-reviewed empirical studies published from 1 January 2013 onward and written in English were eligible for the core synthesis.

The electronic search was conducted in PubMed/MEDLINE, Scopus, Web of Science Core Collection, Embase, APA PsycINFO via EBSCOhost, and CINAHL via EBSCOhost. The final search architecture replaced Emcare with CINAHL to strengthen nursing, allied-health, and service-pathway coverage. Separate search strategies were developed for two thematic streams—sexual violence/sexual assault and domestic violence/IPV—and, within each stream, a core search and a supplementary mental health-focused search were applied. The strategy deliberately balanced sensitivity and specificity by anchoring violence concepts in title or major-topic fields while broadening the second concept block to include follow-up, continuity, advocacy, case management, telehealth, safety planning, and care-pathway terms. This title- or major-topic anchoring was used to reduce retrieval of records in which violence was incidental rather than the central population or service context. The trade-off was a possible reduction in sensitivity for studies using broader service-delivery terminology or alternative violence labels; this limitation is addressed below. Search strategies were adapted to the syntax of each database and are reported in full in Supplementary File S1.

Across the 24 database-query runs, the final search identified 9788 records. After deterministic deduplication within the two thematic search streams, 3944 records remained. After cross-stream consolidation, 3708 unique records were retained for title/abstract screening. At this stage, 3624 records were excluded because they did not meet the Population–Concept–Context criteria, focused only on acute/emergency/forensic or first-contact care, described screening, hotline, referral-only, awareness, or service-access studies without an identifiable post-acute continuity mechanism, had an ineligible publication type, or involved an irrelevant population, setting, or concept. Deduplication was performed in a structured spreadsheet workflow using DOI where available, followed by normalized-title matching and manual verification of near-duplicates.

Before full screening, the two reviewers calibrated the operational post-acute boundary on a mixed subset of records from both the sexual violence and IPV streams. Borderline records included emergency, forensic, screening, referral-only, hotline, and loosely defined support interventions. Studies were retained only when they described an identifiable continuity mechanism beyond the initial emergency, forensic, screening, hotline, or first-contact phase, such as scheduled medical or psychological follow-up, ongoing advocacy or case management, safety-contact calls, outreach, telehealth follow-up, structured therapy, or longer-term stabilisation support. Records were excluded when they were limited to acute examination, first-line crisis response, screening, awareness, hotline contact, or passive referral without evidence of organised subsequent contact. Title/abstract screening and full-text assessment were then performed independently by two reviewers using predefined eligibility criteria; disagreements were resolved by consensus, with involvement of a third reviewer when needed.

Reports were retained as core studies when they described an identifiable post-acute intervention, follow-up pathway, continuity mechanism, or recovery-oriented model. Additional background, guidance, and contextual literature was used only where directly relevant to frame the introduction and discussion. These sources were not part of the charted scoping-review corpus, were not counted as included studies, and did not contribute to the construction of model clusters or to claims about intervention effectiveness.

Data charting was performed using a standardised extraction framework that captured author, year, country, study design, population, type of violence, entry point into the pathway, operational definition/timing of the post-acute phase, intervention components, professionals involved, setting, duration/intensity of follow-up, continuity mechanisms, reported outcomes, barriers/facilitators, and relevance for the Italian context. The data-charting form was piloted on an initial subset of studies representing both violence streams and then refined before full extraction. Charting was performed independently by two reviewers and reconciled through discussion to ensure consistency in category assignment, particularly for multi-component interventions and for the operational boundary between first-contact services and post-acute continuity pathways. The principal characteristics of included studies are summarized in Table 1; the populated data-charting matrix for the 44 core studies is provided in Supplementary Table S4, including post-acute entry point, operational timing, duration/intensity, continuity mechanism, and follow-up horizon.

Findings were synthesised narratively and comparatively. Included core studies were first described by country, design, violence type, and setting, and were then grouped into broad organisational categories, including trauma-focused therapeutic models, advocacy and case-management models, sexual assault care centre pathways, hospital- or clinic-based interventions, digital/telehealth models, housing and stabilisation models, outreach and safety-contact models, and programmes for system-involved survivors. Categories were derived iteratively from the charted intervention components and continuity mechanisms, while allowing multi-component studies to inform more than one interpretive category where appropriate. The four comparative dimensions used in the cross-country synthesis (temporal structuring, coordinating or relational function, cross-sector integration, and setting/delivery architecture) crystallised during iterative charting and narrative synthesis after extraction of entry point, timing, intervention components, workforce, setting, continuity mechanisms, and follow-up horizon; they were therefore used as an interpretive synthesis structure rather than imposed as a priori service categories. No meta-analysis was planned because of substantial clinical, methodological, and organisational heterogeneity. Formal risk-of-bias assessment was not used as a basis for excluding studies from the core synthesis, because the purpose of the review was to map and compare the range of available models rather than to pool effect estimates.

**Figure 1:**
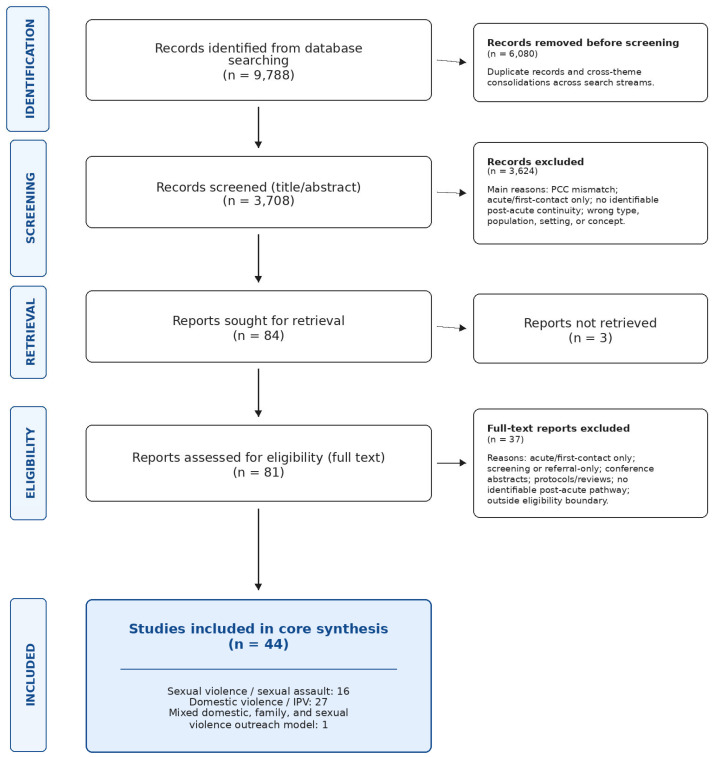
PRISMA-ScR flow diagram of study selection, including aggregate reasons for title/abstract exclusions.

Selected grey literature was used only to support conceptual framing, service-model interpretation, and contextual discussion, especially in relation to the Italian setting. This scoping review was conducted in accordance with the JBI methodological guidance for scoping reviews and reported according to the Preferred Reporting Items for Systematic Reviews and Meta-Analyses extension for Scoping Reviews (PRISMA-ScR). The completed PRISMA-ScR checklist is provided in Supplementary File S2 and as a separate reporting checklist file, and the study-selection process is shown in the PRISMA-ScR flow diagram in Figure 1.

## 3. Results

### 3.1. Study Selection

After database searching, deduplication, title/abstract screening, and full-text assessment, 44 studies met the inclusion criteria for the core synthesis. Of these, 16 focused on sexual violence/sexual assault, 27 focused on domestic violence/intimate partner violence (IPV), and one addressed a mixed domestic, family, and sexual violence (DFSV) primary-care outreach model. The study-selection flow and aggregate reasons for title/abstract exclusions are shown in Figure 1.

Of the 81 reports assessed in full text, 37 were excluded from the core synthesis. These reports were generally removed because they focused primarily on the acute/emergency phase, first forensic examination, early crisis management, or service access without a sufficiently identifiable follow-up component. Other exclusions concerned conference abstracts, protocols, reviews, or studies describing support in overly general terms without a recognisable post-acute continuity pathway. One newly identified report could not be retrieved in full text and one newly retrieved conference abstract was excluded from the core synthesis for lack of sufficient full-text data.

The final six-database search architecture increased nursing/allied-health coverage through CINAHL and identified three additional core studies relevant to follow-up attendance, outreach, and safety-contact continuity [18,19,20].

### 3.2. General Characteristics of Included Studies

The final core sample was methodologically heterogeneous and included randomised–controlled trials, pilot trials, quasi-experimental studies, mixed-methods evaluations, retrospective observational studies, qualitative studies, and service evaluations. The evidence base was predominantly U.S. based, although relevant studies were also identified from Australia, Belgium, Brazil, Canada, Finland, Hong Kong, Mexico, the Netherlands, Portugal, South Africa, Sweden, and the United Kingdom [19,20,21,22,23,24].

The sexual violence literature was comparatively smaller and more concentrated around a limited number of intervention types, especially trauma-focused psychotherapies, sexual assault care centres, medical follow-up, follow-up attendance, and digital or telemedicine-based approaches [18,22,25,26,27]. By contrast, the IPV literature was broader and more organisationally diverse, including psychotherapy, advocacy, housing-first models, nurse-led or clinic-based services, outreach and safety-contact programmes, digital tools, and interventions for system-involved survivors [19,20,28,29,30,31].

For transparency, the principal characteristics and continuity mechanisms of the 44 core studies are summarized in Table 1, while the more detailed populated charting matrix is provided in Supplementary Table S4. Because the post-acute entry point ranged from early scheduled review after acute or forensic care to flexible outreach, structured therapy, and 6- to 24-month housing or stabilisation interventions, Supplementary Table S4 reports the entry point, operational timing, continuity mechanism, and follow-up horizon for each included study.

### 3.3. Conceptualisation of the Post-Acute Phase

One of the clearest findings was the lack of terminological consistency in how the post-acute phase was described. Only some papers explicitly used terms such as follow-up, aftercare, continuity of care, or care pathway. In many cases, the post-acute dimension had to be inferred from the intervention structure rather than from standardised terminology. This was particularly evident in studies of post-rape services, where mental health support, follow-up attendance, and later engagement were embedded in broader service trajectories rather than presented as a formally distinct phase [21,23,37].

A similar pattern emerged in the IPV literature. Many studies did not label their interventions as post-acute pathways, yet clearly addressed needs arising after the first disclosure, shelter entry, court referral, or healthcare contact. This was the case for advocacy-based recovery models, co-located mental health services, and structured community interventions designed to operate beyond the immediate crisis response [31,53,57,59].

Overall, the included studies suggest that the post-acute phase is often present in practice even when it is not explicitly named as such. For this reason, the review relied on an operational definition centred on continuity, structured follow-up, coordinated support, or recovery-oriented intervention delivered beyond the first acute contact.

The heterogeneity was therefore temporal as well as terminological. Some studies entered the post-acute phase very soon after acute rape care through scheduled medical or counselling follow-up, whereas others examined medium-term safety-contact or psychoeducational programmes, flexible outreach, digital continuity, or 6-, 12-, and 24-month housing and stabilisation models [18,20,29,37,49,50,51]. This range reinforced the need to apply the post-acute boundary through the presence of an identifiable continuity mechanism rather than through a uniform time cut-off.

### 3.4. Post-Acute Intervention Models After Sexual Violence

The 16 sexual violence studies clustered around four broad models.

The first consisted of trauma-focused or early psychological interventions delivered after sexual assault. These included prolonged exposure, cognitive processing therapy, one-session PTSD-oriented treatment, and online therapist-facilitated interventions [25,26,34,35]. Together, these studies show that the immediate post-assault period has frequently been conceptualised as a window for targeted trauma treatment rather than for medical assessment alone.

A second group of studies examined service organisation within sexual assault care centres or post-rape care systems. The Belgian and Dutch studies were particularly useful in this regard, as they described what care was actually offered after the initial contact and how survivors engaged with professional services over time [22,23]. The South African appraisal highlighted that mental-health support was central to post-rape care services but unevenly available [21].

A third model concerned medical follow-up, survivor retention, and loss to follow-up after acute care. The Australian retrospective study on follow-up attendance and the U.S. study of rape victims seen in acute medical care illustrated a recurring problem in this area, namely that recommended post-assault review does not automatically translate into actual service uptake [18,37]. In the U.S. study, only 28% of rape victims attended the recommended medical/counselling follow-up, and disability, current mental illness, public assault location, prior mental health history, completed SANE examination, and available social support were all associated with attendance patterns [18]. Relatedly, work on advocacy engagement suggested that continued contact with services may be shaped by changing service conditions, including the disruptions associated with the COVID-19 period [38].

The fourth model involved digital, mobile, or telemedicine-supported continuity interventions. These ranged from text-messaging support and mobile health pilot interventions to telemedicine-assisted follow-up after sexual assault and IPV [27,36,39,40]. Although heterogeneous in design, these studies collectively suggest that remote continuity tools may reduce barriers to follow-up and help sustain post-assault engagement when in-person pathways are difficult to maintain.

Overall, the sexual violence literature portrayed post-acute care as a combination of early trauma intervention, centre-based service provision, follow-up attendance efforts, and emerging digital continuity models. Compared with the IPV literature, however, it was less developed in terms of broader long-term social or multidimensional recovery pathways.

### 3.5. Post-Acute Intervention Models After Domestic Violence/IPV

The 27 IPV/domestic violence studies, together with one mixed domestic, family, and sexual violence outreach model, showed substantially broader organisational diversity than the sexual violence literature.

A first major subgroup comprised psychotherapeutic and psychosocial interventions. These included psychological advocacy, trauma-focused therapy in shelters, group interventions, compassion-based therapy, culturally tailored interventions, qigong-based mental health support, and internet-delivered CBT [28,41,42,43,44,45,46,47]. Although their formats varied, these studies consistently addressed mental health recovery, coping, and empowerment after the acute phase.

A second major subgroup concerned advocacy and case-management-oriented models. PATH represented one of the clearest examples of advocacy embedded in a domestic violence service setting [28], while other studies linked advocacy more directly to recovery, service access, and hospital-based interventions [53,55,56]. The digital warm handoff model extended this logic by attempting to improve linkage from emergency care to advocacy support [54].

A third subgroup focused on housing and trauma-informed stabilisation models. The Domestic Violence Housing First studies provided one of the strongest examples of a longer-term post-acute pathway, with findings reported at 6, 12, and 24 months [29,49,50]. This line of work was further strengthened by evidence suggesting that trauma-informed practices and housing support may have separate and cumulative effects on safety, housing stability, and mental health over time [51].

A fourth group involved digital and eHealth interventions. These included MySteps and the SAFE eHealth intervention, both of which suggest that digital tools may operate as structured post-acute resources for safety, support, and recovery, rather than as information delivery alone [48,60]. In the IPV literature, digital interventions therefore appeared as plausible components of continuity pathways, although their safety, acceptability, and effectiveness remain context-dependent.

A fifth subgroup concerned nurse-led, clinic-based, co-located, and outreach service models. The Mexico City trial showed that a nurse-delivered clinic-based intervention could be embedded within routine healthcare [24], while other studies described community-based nurse-led domestic violence services, co-location of specialised mental health care within advocacy settings, and multidisciplinary primary-care outreach for women and children affected by domestic, family, and sexual violence [19,30,57]. These models are especially relevant because they illustrate how post-acute care can be integrated into existing service infrastructures rather than delivered only through highly specialised standalone programmes.

A sixth subgroup involved interventions for system-involved survivors, particularly women mandated to services through child protection or court systems. The Parenting and Safety Program and the MOVE intervention are important in this regard because they addressed safety, parenting, and mental health in populations with complex social and institutional vulnerabilities [31,61]. These studies broaden the concept of post-acute care by showing that continuity pathways may also need to respond to legal, parenting, and system-involvement dimensions.

Taken together, the IPV and mixed DFSV literature described post-acute care as a broad continuum that included therapy, advocacy, housing support, digital tools, healthcare-linked interventions, outreach, safety-contact programmes, and specialised programmes for highly vulnerable groups.

### 3.6. Cross-Country Comparison of Organisational Features

Despite substantial heterogeneity, several dimensions allowed meaningful comparison across countries and service systems.

A first dimension was the degree to which follow-up was structured in time. Some interventions were clearly scheduled and protocolised, with fixed session numbers or predefined follow-up intervals, as seen in psychotherapy trials, housing-first evaluations, and safety-contact follow-up [20,29,41,47,50]. Others were more flexible or service-led, with continuity depending on organisational capacity, outreach, and survivor engagement rather than on a fixed schedule [19,23,38,53].

A second dimension concerned the presence of an explicit coordinating or relational function. In the IPV literature, this role was often fulfilled by advocacy, case-management, outreach, or safety-contact models [19,20,28,53,55], whereas in the sexual violence literature it appeared more variably through centre-based pathways, engagement efforts, SANE-linked follow-up, or post-examination support mechanisms [18,22,36,38]. This suggests that one of the most important cross-system distinctions lies in whether some actor remains responsible for maintaining continuity after the first contact.

A third comparative dimension was the level of integration across sectors. Some pathways remained mainly confined to one domain, such as psychotherapy or medical follow-up [18,26,37,46], whereas others linked health, mental health, social support, housing, advocacy, and community outreach more explicitly [19,29,51,57]. The IPV and mixed DFSV literature more often displayed this broader multi-sector integration.

A fourth dimension concerned the setting of care. Post-acute pathways were delivered across sexual assault centres, shelters, outpatient clinics, hospitals, primary-care outreach services, community organisations, digital platforms, and mixed or hybrid service environments [19,21,30,40,56]. This indicates that continuity is not tied to a single institutional location, but can be organised through a range of entry points and service architectures.

To facilitate comparison across settings, the main organisational models identified in the review and their recurrent strengths, gaps, and implications are synthesized in Table 2.

Three examples illustrate how the four dimensions cut across individual studies rather than functioning as mutually exclusive model labels. Darnell et al. [18] combined a clearly defined temporal element (recommended post-assault medical/counselling follow-up after acute rape care) with a retention problem, because only a minority attended the recommended appointment. The Domestic Violence Housing First studies [29,49,50] mapped simultaneously onto temporal structuring, cross-sector integration, and material stabilisation because they combined advocacy and housing support over 6-, 12-, and 24-month horizons.

### 3.7. Reported Outcomes, Barriers, Facilitators, and Recurrent Service Gaps

The reported outcomes were highly heterogeneous. Clinical and psychological outcomes included PTSD symptoms, depression, distress, self-compassion, trauma-related symptoms, and broader mental health indicators [25,34,44,45]. Service-related outcomes included follow-up attendance, acceptability, feasibility, service uptake, linkage, and engagement with advocacy or digital support [18,19,27,37,39,54]. Social and recovery-related outcomes included safety, housing stability, wellbeing, empowerment, perceived safety, and, in some studies, parenting-related change [20,29,31,51,61].

Identifiable support models emerged in both streams, but they were accompanied by persistent gaps. In sexual violence, recurring models included trauma-focused early intervention, sexual assault centre-based pathways, post-assault follow-up, retention-oriented linkage, and digital continuity approaches [18,22,25,37,39]. In IPV and mixed DFSV, recurring models included psychotherapy, advocacy, housing-first stabilisation, nurse-led or clinic-based care, outreach and safety-contact programmes, digital interventions, and structured programmes for system-involved survivors [19,20,28,29,30,31].

Several barriers recurred across the core literature. Fragmentation after first contact was common, with survivors not always transitioning from entry-point services to sustained support [18,37,58]. Follow-up may be recommended but not attended, advocacy may not be systematically offered, and survivors may experience pathways as uneven, difficult to access, or insufficiently coordinated [18,37,58,59]. Other barriers included uneven access to mental health support, weak coordination, accessibility and acceptability obstacles, and difficulties maintaining engagement with routine or specialist services after violence exposure [21,59].

The most recurrent facilitators were scheduled re-contact, advocacy or case-management functions, practical support enabling participation, trauma-informed care, safety-contact strategies, outreach, and service models able to integrate multiple domains of need [19,20,28,31,52,57]. Telemedicine and other digital approaches also emerged as potential continuity tools, particularly where in-person retention may be difficult [27,40,48]. Taken together, these findings indicate that loss after first contact is also an organisational and system-design problem, not simply a matter of individual survivor choice.

## 4. Discussion

This review mapped post-acute care pathways for survivors of sexual violence and domestic violence/IPV across different countries and service systems. The findings suggest that the post-acute phase is increasingly recognised in practice, but remains inconsistently defined and unevenly formalised in the literature [18,21,22,28]. Read alongside major international guidance, this heterogeneity reflects a deeper tension between systems-centred care and survivor-centred care: services are often organised around institutional entry points, whereas survivors may define recovery in broader and more relational terms [2,4,5,7].

Trauma-and-violence-informed care extends standard trauma-informed care by shifting attention from individual trauma symptoms alone to the structural and relational conditions that shape access, trust, safety, and recovery [3,9]. In post-acute pathways, this means that continuity should not be evaluated only by whether referral or treatment was offered, but also by whether services reduce stigma, avoid retraumatization, address intersecting barriers such as poverty, migration status, disability, language, and regional service availability, and create organisational conditions in which survivors can safely re-engage over time. From this perspective, loss to follow-up may reflect not only individual non-attendance, but also service fragmentation, unsafe contact procedures, inaccessible delivery models, or insufficient coordination across agencies.

The four comparative dimensions identified in the Results—temporal structuring, coordinating or relational functions, cross-sector integration, and setting/delivery architecture—provide a useful framework for interpreting this evidence. They also clarify an important difference between the sexual violence and IPV studies. The sexual violence literature was smaller and more concentrated around early trauma-focused interventions, sexual assault care centre pathways, post-assault follow-up, retention after acute care, and digital continuity tools [18,22,25,26,39]. The IPV literature was more organisationally expansive, incorporating psychotherapy, advocacy, housing-first interventions, clinic-based and co-located services, outreach and safety-contact programmes, digital approaches, and tailored pathways for system-involved survivors [19,20,28,29,31,57].

First, temporal structuring makes continuity visible and measurable. Some included interventions used scheduled sessions, predefined follow-up intervals, safety-contact calls, or fixed programme lengths [20,31,41,47]. Others were intentionally flexible, with continuity depending on organisational capacity, outreach, advocacy, or survivor engagement rather than on a fixed schedule [19,23,38,53]. For Italy, this distinction supports evaluating explicit follow-up timelines while allowing regional services and anti-violence networks to adapt intensity and timing to survivor needs and local feasibility [5,15,16].

Second, the coordinating or relational function appears central to whether referral becomes actual connection. In the IPV literature, advocacy was one of the most recurrent organisational components, appearing in psychological advocacy models, hospital-based domestic violence interventions, outreach services, safety-contact programmes, and linkage strategies from emergency settings to longer-term support [19,20,28,54,56]. Several studies pointed to the value of a dedicated person or function that helps survivors navigate fragmented systems, maintain contact, and translate referral into engagement [52,53,57]. Recent synthesis work and service specifications similarly suggest that warm referral models, co-location, and hospital-community bridging strategies may reduce loss between services [10,62,63,64]. Provider-focused intake work also emphasizes that service matching depends on understanding survivors’ goals, trauma histories, and health status [65].

Third, cross-sector integration is especially important because post-acute needs are rarely limited to a single clinical domain. Interventions limited to one component may be useful, particularly when they are well-designed psychotherapeutic programmes [42,46]. However, the most structurally relevant models linked safety, mental health, practical support, housing, advocacy, and longer-term stabilisation [29,31,51]. This is particularly evident in IPV, where housing security, environmental stability, privacy, control, predictability, and a felt sense of home may affect safety and recovery trajectories [50,66,67]. Sexual violence and IPV pathways should therefore remain distinct but interoperable: post-sexual-assault care may require timely trauma-focused support, mental health referral, and follow-up after the medico-legal phase, whereas IPV pathways may more often require chronic safety management, advocacy, housing, and longer-term psychosocial stabilisation [18,20,22,25,29,51].

Fourth, setting and delivery architecture shape whether continuity is accessible in practice. Post-acute pathways may be organised through sexual assault centres, hospitals, shelters, primary care, community-based services, telehealth, digital tools, or hybrid models [19,21,30,40,56]. The core evidence included text messaging, mHealth, telemedicine, internet-delivered therapy, and eHealth models in both the sexual violence and IPV studies [27,39,40,47,48,60]. Contextual literature adds that digital capacity expanded rapidly during COVID-19 across violence services, but often as organisational adaptation rather than as an evaluated pathway [68]. Survivor co-design work further indicates that the usability and accessibility of digital information resources should be treated as design requirements, not as secondary presentation issues [69]. Remote modalities may therefore support engagement when ordinary pathways are difficult to complete, but only where contact modalities are survivor controlled, safe from perpetrator monitoring, accessible to survivors with limited digital resources, supported by cybersecurity and safe-documentation procedures, and accompanied by clear non-digital alternatives [62,70].

These dimensions also expose persistent gaps and equity issues. Several core studies suggested that follow-up is often recommended without being reliably achieved, that service transitions remain vulnerable points, and that substantial underutilisation persists even when services formally exist [18,21,37,58]. Adjacent evidence on low uptake of gynecological consultation after domestic or sexual violence during pregnancy follow-up, help-seeking among Bosnian women in shelters, Chinese immigrant IPV survivors’ experiences, and pandemic-era resource utilization all indicate that formal availability does not guarantee usable continuity [71,72,73,74]. In addition, the recent British Columbia Healthy Connections Project trial provides adjacent evidence that nurse-home visiting may influence IPV-related maternal outcomes over a 24-month postpartum horizon; it was treated as contextual evidence rather than added to the charted core synthesis because it was not primarily designed as a post-acute violence pathway after first contact [75]. Recent work on immigrant and minority survivors further shows that intersectional disadvantage can affect access, trust, uptake, and the perceived safety of formal services [64].

For the Italian context, transferability should be understood as heuristic rather than directly demonstrated. The DPCM 24 November 2017 and the related Percorso per le donne che subiscono violenza provide a national healthcare-oriented frame for emergency and socio-health assistance, while Codice Rosa/Pink Code experiences, anti-violence centres, territorial services, and regional health-system arrangements shape how continuity is implemented in practice [12,13,14,15,16]. The 2025–2027 National Strategic Plan further emphasizes coordinated, multi-level action, monitoring, and integration between institutional actors and anti-violence networks [13]. The relevant implementation question is therefore whether these existing architectures generate observable post-acute continuity after first access: structured re-contact, warm handoffs, identifiable coordination, psychological and advocacy follow-up, social stabilisation, and survivor-defined outcome monitoring. The four-dimensional framework therefore provides local evaluation questions rather than a ready-made imported model.

Provisional evidence-informed organisational directions for post-acute follow-up.

For ease of reference, the principal evidence-informed local evaluation questions emerging from the synthesis are reorganised in Box 1 around the same four dimensions used for cross-country comparison. These questions are provisional implications for local evaluation, not prescriptive service standards.

Box 1 **Four-dimensional framework for locally evaluating post-acute follow-up pathways.**

**Organisational Dimension**

**Local Evaluation Question**
Temporal structuring—phased follow-up timelineDo local pathways specify early re-contact, consolidation, and medium-term stabilisation phases, while allowing flexible timing when fixed scheduling is unsafe or infeasible?Temporal structuring—minimum offer and graded intensityDo local pathways define a minimum post-acute offer, such as safety review, psychological needs assessment, practical service information, and scheduled re-contact, with intensity adjusted to clinical, psychosocial, safety, and material vulnerability?Coordinating function—survivor navigator/care coordinatorIs there an identifiable coordinating function responsible for safe contact, monitoring uptake, and bridging health, psychological, social, and advocacy services?Coordinating function—warm handoffDo local pathways reduce passive referral through active transfer processes in which the receiving service is contacted and linkage is facilitated within appropriate confidentiality safeguards?Cross-sector integration—material stabilisation in IPV pathwaysDo local IPV pathways evaluate whether housing, economic, territorial, and social supports are needed, recognizing that recovery is closely tied to safety and stability?Cross-sector integration—post-sexual-assault care beyond medico-legal prioritiesDo local post-sexual-assault pathways evaluate psychological follow-up, advocacy, and engagement monitoring after the forensic/medical phase?Cross-sector integration—survivor-defined outcome measurementAre local evaluations designed to track not only symptoms and attendance, but also safety, empowerment, continuity, acceptability, and survivor-defined healing?Setting and delivery architecture—hybrid in-person and remote follow-upCan face-to-face care be safely combined with telemedicine, phone, or secure digital tools where survivor-controlled contact, privacy safeguards, digital inclusion, and non-digital alternatives are available?Setting and delivery architecture—co-design, feedback loops, and quality monitoringAre survivor feedback, service evaluation, collaborative quality improvement, and, where appropriate, human-supervised digital or AI-assisted audit tools used to revise pathways over time?

The questions summarized in Box 1 should be read as provisional implications derived from a heterogeneous evidence map, not as clinical standards. The four dimensions do not define a single model; rather, they identify elements that can be specified, implemented, measured, and revised locally.

A temporal dimension allows services to make follow-up observable through early re-contact, consolidation, and medium-term stabilisation, while retaining flexibility for survivors whose safety, mobility, or service access changes over time [18,19,20,29,31,41]. A coordinating dimension highlights the potential value of advocacy, case management, outreach, safety-contact, or equivalent survivor-navigation roles that convert referral into actual connection and reduce loss after first access [19,20,28,53,55].

A cross-sector dimension recognizes that post-acute needs may include psychological care, safety planning, advocacy, housing, material stabilisation, and legal or system-involved support [18,20,22,25,29,51]. A setting and delivery dimension recognizes that continuity can be organised through centres, hospitals, shelters, primary care, community services, and hybrid in-person or remote models, provided that digital safety and access barriers are actively managed [27,40,48].

Finally, the same framework can support pathway-level service evaluation. Indicators might include attendance, missed follow-up, time to first re-contact, successful warm handoff, uptake of psychological, advocacy, or social support, safety-review completion, survivor-reported acceptability, empowerment, perceived safety, and continuity. Equity monitoring could examine differential loss to follow-up by geography, migration status, disability, language barriers, age, or service-access constraints. Where appropriate, human-supervised digital or AI-assisted audit tools could support dashboarding, detection of recurrent missed handoffs, monitoring of service delays, and summarisation of anonymised service-use patterns. Such tools should remain a quality-improvement layer, not an automated decision-maker or substitute for survivor-centred care or professional judgement. Any future digital or AI-assisted audit should rely on fully anonymised or aggregated administrative data whenever possible, avoid identifiable free-text extraction, and include explicit assessment of re-identification risks, particularly where text mining or natural language processing is applied to sensitive service records. Governance should include data minimisation, confidentiality, trauma-informed oversight, bias monitoring, cybersecurity, non-discrimination, survivor safety, and avoidance of punitive or surveillance-oriented use.

## 5. Strengths and Limitations

Several limitations should be considered. First, the post-acute phase was described inconsistently across the literature, which required an operational rather than purely terminological approach to eligibility. The post-acute boundary was therefore applied through the presence of an identifiable continuity mechanism beyond first contact, but some judgement remained necessary for borderline studies. Second, no external protocol was registered, although an internal review plan defined the review questions, eligibility criteria, search architecture, screening rules, and charting variables before full screening. Third, the search strategy involved an intentional specificity/sensitivity trade-off. By anchoring violence concepts in title or major-topic fields in several databases, we reduced retrieval of records in which violence was incidental, but may have missed studies using broader service-delivery terminology, alternative violence labels, or non-English terminology. Fourth, the review was limited to English-language peer-reviewed empirical studies from 2013 onward. This restriction is particularly relevant for transferability to Italy and Europe because governmental pathway documents, regional service evaluations, anti-violence centre reports, NGO evaluations, and non-English Italian, French, Spanish, or other European publications may describe operational models not represented in the indexed English-language literature. Grey literature was therefore used only for contextual framing and Italian service interpretation, not as part of the charted core evidence base. Fifth, no formal critical appraisal was used to exclude studies, and the findings should therefore be interpreted as a mapping of models and reported outcomes rather than as comparative effectiveness evidence. Sixth, some model clusters were represented by repeated outputs from related intervention families, particularly in housing-first research. Finally, this review should be interpreted as a thematic international mapping of post-acute pathway models rather than as a formal country-by-country comparative health-systems evaluation.

Despite these limitations, the review supports a clear overall interpretation: the quality of responses to sexual violence and domestic violence/IPV depends on whether services organise explicit continuity mechanisms after first contact. For Italy, the findings provide evidence-informed questions for service organisation—rather than a ready-made imported model—concerning temporal structuring, warm handoffs, survivor navigation, cross-sector integration, hybrid delivery, and links between healthcare, advocacy, and social stabilisation.

## 6. Conclusions

This review indicates that post-acute care after sexual violence and domestic violence/IPV is best examined through the continuity mechanisms that translate initial contact into sustained recovery support. Across the mapped literature, these mechanisms varied across violence types and service systems, but could be interpreted through four organisational dimensions: temporal structuring, coordinating or relational functions, cross-sector integration, and setting or delivery architecture.

For Italy, the mapped findings do not support importing a single model. Instead, they support locally evaluating whether existing national, regional, healthcare, anti-violence, and territorial service architectures provide visible post-acute continuity through structured re-contact, warm handoffs, identifiable coordination, integrated psychological, advocacy, and social support, safeguarded hybrid delivery, survivor-defined outcome measurement, and quality-improvement feedback loops capable of monitoring whether pathways work as intended.

## Figures and Tables

**Table 1:** Summary characteristics and continuity mechanisms of included core studies.

Study	Country	Violence Type	Setting/Entry Point	Model/Post-Acute Component	Continuity Mechanism	Follow-Up Horizon
Darnell et al. (2015) [18]	USA	Sexual violence	Emergency/acute medical care; SANE-linked follow-up	Scheduled medical/counselling follow-up after acute rape care	Scheduled follow-up appointment and linkage to medical/mental health services	Recommended post-assault follow-up; attendance assessed after acute visit
Triandafilidis et al. (2025) [19]	Australia	Mixed DFSV	Multidisciplinary primary-care outreach service	Proactive, trauma-informed primary-care outreach with advocacy and multidisciplinary care	Flexible outreach, trusted provider contact, multidisciplinary communication, and facilitated linkage	Feasibility/acceptability assessment during service pilot
Westwood et al. (2020) [20]	Australia	IPV/domestic	Integrated domestic and family violence programme; women’s safety service	Safety-contact programme linked to integrated perpetrator intervention	Telephone safety contact, emotional support, practical safety planning, and survivor-centred monitoring	Approximately 10 weeks of safety-contact support
Abrahams et al. (2017) [21]	South Africa	Sexual violence	Post-rape care services	Integrated mental health support within post-rape services	Centre-based psychosocial/referral coordination	Cross-sectional service snapshot
Baert et al. (2021) [22]	Belgium	Sexual violence	Sexual Assault Care Centres	Integrated multidisciplinary sexual assault centre model	Centre-based psychosocial/referral coordination	Service pathway description
Bicanic et al. (2014) [23]	Netherlands	Sexual violence	Sexual assault centre	Integrated centre with professional follow-up/referral	Centre-based psychosocial/referral coordination	Service utilization after assault
Gupta et al. (2017) [24]	Mexico	IPV/domestic	Clinic-based care	Nurse-delivered clinic intervention	Embedded healthcare follow-up and referral support	Trial follow-up
Foa et al. (2013) [25]	USA	Sexual violence	Therapy/outpatient mental health	Trauma-focused psychotherapy	Structured therapy sessions and assessment	Post-treatment follow-up
Nixon et al. (2016) [26]	Australia	Sexual violence	Psychotherapy service	Early cognitive processing therapy	Structured therapy sessions and assessment	Short-term follow-up
Fagen et al. (2025) [27]	Canada	Sexual violence	Emergency department linked virtual follow-up	Telemedicine-enabled clinical follow-up	Remote/digital contact or telehealth follow-up	7-year service investigation
Ferrari et al. (2018) [28]	United Kingdom	IPV/domestic	Domestic violence service setting	Psychological advocacy	Advocacy/case-management navigation	Post-intervention follow-up
Sullivan et al. (2023) [29]	USA	IPV/domestic	Housing + advocacy services	Housing First + survivor-centred advocacy	Longitudinal housing/stabilisation support	24 months
Hollingdrake et al. (2025) [30]	Australia	IPV/domestic	Community-based nurse-led service	Nurse-led domestic violence service	Embedded healthcare follow-up and referral support	Service-use perspectives
Rizo et al. (2018) [31]	USA	IPV/domestic	Community-based group intervention	13-week psychoeducational safety-parenting-mental health program	Structured therapy sessions and assessment	3 and 6 months
Miller et al. (2015) [32]	USA	Sexual violence	Post-assault care/forensic nursing context	Brief psychoeducational video intervention	Structured post-contact follow-up/service-use tracking	Short-term follow-up
Walsh et al. (2017) [33]	USA	Sexual violence	Post-assault intervention	Video-based behavioural prevention intervention	Structured post-contact follow-up/service-use tracking	Follow-up after recent assault
Rajan et al. (2022) [34]	Sweden	Sexual violence	Post-assault psychological treatment	Single-session PTSD intervention	Structured post-contact follow-up/service-use tracking	Short-term follow-up
Littleton et al. (2016) [35]	USA	Sexual violence	Online therapist-facilitated program	Digital trauma-focused therapy	Remote/digital contact or telehealth follow-up	Post-program follow-up
Hicks et al. (2017) [36]	USA	Sexual violence	Post-exam follow-up program	SMS follow-up/engagement support	Structured post-contact follow-up/service-use tracking	Immediate post-examination period
Healey et al. (2023) [37]	Australia	Sexual violence	Medical follow-up after assault	Scheduled medical follow-up pathway	Structured post-contact follow-up/service-use tracking	Attendance at follow-up visit
Engleton et al. (2022) [38]	USA	Sexual violence	Advocacy services	Advocacy-based support	Advocacy/case-management navigation	Pandemic-period service engagement
Dworkin et al. (2023) [39]	USA	Sexual violence	mHealth early intervention	Digital/mHealth early intervention	Remote/digital contact or telehealth follow-up	Early follow-up
Mercier et al. (2024) [40]	Canada	Sexual violence	Telemedicine/virtual care	Telehealth follow-up model	Remote/digital contact or telehealth follow-up	Post-acute virtual care pathway
Johnson et al. (2020) [41]	USA	IPV/domestic	Shelter	Shelter-based psychotherapy	Structured therapy sessions and assessment	Post-treatment follow-up
Santos et al. (2017) [42]	Portugal	IPV/domestic	Group program	Group psychosocial intervention	Structured therapy sessions and assessment	Post-program evaluation
Naismith et al. (2021) [43]	Colombia	IPV/domestic	Group therapy	Compassion-based group therapy	Structured therapy sessions and assessment	Pilot follow-up
Li et al. (2024) [44]	USA	IPV/domestic	Community-based support/therapy	Self-compassion/empowerment intervention	Structured therapy sessions and assessment	Pilot follow-up
Cheung et al. (2019) [45]	Hong Kong	IPV/domestic	Community intervention	Mind–body/Qigong intervention	Structured therapy sessions and assessment	Trial follow-up
Foschiera et al. (2024) [46]	Brazil	IPV/domestic	Psychotherapy service	Psychotherapy protocol	Structured therapy sessions and assessment	Follow-up study
Andersson et al. (2021) [47]	Sweden	IPV/domestic	Internet-based treatment	Internet-delivered CBT	Remote/digital contact or telehealth follow-up	Pilot follow-up
Sabri et al. (2024) [48]	USA	IPV/domestic	Digital safety support	Digital safety and support intervention	Remote/digital contact or telehealth follow-up	Preliminary efficacy follow-up
Sullivan et al. (2023) [49]	USA	IPV/domestic	Housing + advocacy services	Housing First + advocacy	Longitudinal housing/stabilisation support	6 months
Sullivan et al. (2023) [50]	USA	IPV/domestic	Housing + advocacy services	Housing First + advocacy	Longitudinal housing/stabilisation support	12 months
Nnawulezi et al. (2025) [51]	USA	IPV/domestic	Housing/trauma-informed services	Trauma-informed practices + housing intervention	Longitudinal housing/stabilisation support	24 months
Rodgers et al. (2017) [52]	USA	IPV/domestic	Urban community outreach	Community health worker outreach	Structured post-contact follow-up/service-use tracking	Program feasibility
Trevillion et al. (2014) [53]	United Kingdom	IPV/domestic	Advocacy services	Advocacy for recovery	Advocacy/case-management navigation	Observational follow-up
Brignone et al. (2022) [54]	USA	IPV/domestic	Emergency department to advocacy link	Digital warm handoff to advocacy	Advocacy/case-management navigation	Post-ED linkage
Halliwell et al. (2019) [55]	United Kingdom	IPV/domestic	Hospital-based advocacy	Hospital-based advocacy intervention	Advocacy/case-management navigation	Service evaluation
Dheensa et al. (2020) [56]	United Kingdom	IPV/domestic	Hospital-based advocacy	Hospital-based advocacy intervention	Advocacy/case-management navigation	Implementation evaluation
Berry et al. (2024) [57]	USA	IPV/domestic	Advocacy organisation with co-located mental health care	Co-located specialized mental health services	Advocacy/case-management navigation	Program implementation
Hackenberg et al. (2021) [58]	Finland	IPV/domestic	Primary care emergency rooms	Advocacy referral gap analysis	Advocacy/case-management navigation	Prospective observation
Sorrentino et al. (2021) [59]	USA	IPV/domestic	Mental health care pathway	Survivor perspectives on mental health care	Structured post-contact follow-up/service-use tracking	Not intervention-focused
van Gelder et al. (2023) [60]	Netherlands	IPV/domestic	eHealth/remote support	eHealth intervention	Remote/digital contact or telehealth follow-up	RCT follow-up
Rizo et al. (2016) [61]	USA	IPV/domestic	System-involved survivor program	Parenting and safety program (MOVE-related)	Structured programme contact and safety/parenting support	Post-program follow-up

Abbreviations: DFSV, domestic, family, and sexual violence; IPV, intimate partner violence; SANE, sexual assault nurse examiner.

**Table 2:** Comparative models of post-acute care pathways.

Model	Target Population	Core Components	Strengths	Recurrent Gaps	Relevance for Italy
Trauma-focused psychological follow-up	Sexual assault; IPV/domestic	Brief trauma therapy, structured sessions, symptom-focused follow-up, post-assault or post-crisis monitoring	Can address PTSD/depression early; can be protocolised; adaptable to digital delivery	Often narrow clinical focus; limited survivor-defined outcomes; variable long-term follow-up	Useful for evaluating whether DPCM 24 November 2017 and Percorso Donna pathways include an explicit psychological follow-up offer after emergency or forensic care [12].
Advocacy/case management	Primarily IPV/domestic; some sexual assault	Advocate or coordinator role, care linkage, warm handoff, safety review, survivor navigation	May facilitate engagement, service uptake, and continuity across sectors	Implementation depends on staffing, inter-agency trust, and organisational support	Relevant to assessing whether a named navigator, advocate, or case-management function links healthcare, anti-violence centres, social services, and territorial networks after first access [12,13,14].
Integrated sexual assault centres/post-rape services	Sexual assault	Medical, forensic, psychological, and advocacy elements in one pathway or coordinated network	May reduce fragmentation at entry point; can formalise early follow-up	Psychological follow-up and longer-term continuity often remain uneven	Relevant to strengthening post-assault continuity beyond medico-legal priorities within hospital pathways, Codice Rosa/Pink Code experiences, and regional emergency-care models [12,16].
Telehealth/digital continuity models	Sexual assault; IPV/domestic	Text messaging, mHealth, telemedicine, online therapy, remote check-ins	May support accessibility and retention, especially where in-person follow-up is difficult	Privacy, safety, digital literacy, and digital divide issues remain significant	Potentially relevant for hybrid follow-up within regional networks, provided survivor-controlled contact, privacy safeguards, digital inclusion, and safe fallback in-person options are specified.
Housing-first/stabilisation models	IPV/domestic	Housing support, trauma-informed care, advocacy, longer-term stabilisation	Can address safety, wellbeing, and housing security over time	Evidence concentrated in limited settings; transferability may require local adaptation	Relevant to IPV pathways where anti-violence centres, municipalities, social services, and territorial welfare actors must address safety, housing, and material stabilisation beyond healthcare [13,14].
Nurse-led/clinic-based/co-located services	Mainly IPV/domestic	Embedded support in clinics, community nursing, co-located mental health and advocacy	Can bring follow-up into routine care and may reduce access barriers	May remain service-specific unless coordination with external networks is explicit	Relevant to territorial integration through the Italian National Health Service, community health structures, primary care, and possible co-location with anti-violence or psychological supports [12,13].
System-involved survivor programmes	IPV/domestic	Parenting, safety, mental health, court/CPS-linked supportive programming	Can address complex survivor needs often neglected by standard pathways	Evidence base is still relatively small and context-specific	Useful for high-complexity pathways involving child protection, courts, social services, healthcare, and anti-violence networks, where follow-up intensity may need to be graded [12,13].
Outreach and safety-contact models	Primarily IPV/domestic and mixed DFSV	Proactive outreach, flexible primary-care access, safety-contact calls, practical support, multidisciplinary collaboration	May reduce access barriers, build trust, and support perceived safety after first contact	Evidence remains mostly qualitative or service-specific; sustainability and transferability require evaluation	Relevant to evaluating scheduled re-contact and proactive outreach after emergency access, Codice Rosa/Pink Code activation, anti-violence centre contact, or territorial referral [12,13,14,16].

Similarly, linkage-oriented or hybrid models illustrate how delivery architecture and coordinating functions can overlap: Brignone and Gomez [54] tested a digital warm handoff from emergency care to advocacy, Fagen et al. [27] described a virtual bridge from acute care to follow-up, and Triandafilidis et al. [19] described flexible multidisciplinary outreach designed to build trust and facilitate linkage. These examples show why the framework was used as a comparative interpretive structure rather than as a rigid typology.

## Data Availability

No new data were created or analyzed in this study.

## Supplementary Materials

The following supporting information can be downloaded at: https://www.mdpi.com/article/10.3390/healthcare14121735/s1, Supplementary File S1 contains the full final search strategies, platforms, filters, search dates, and records retrieved by database and query. Supplementary File S2 contains the PRISMA-ScR reporting checklist with manuscript locations. Supplementary File S3 contains the data-charting form and the populated extraction matrix for the 44 core studies, including post-acute entry point, operational timing, continuity mechanism, and follow-up horizon. Supplementary File S4 contains reports excluded from the core synthesis after full-text assessment, with reasons for exclusion.

## Abbreviations

The following abbreviations are used in this manuscript:

AI	artificial intelligence
CBT	cognitive behavioural therapy
CPS	child protective services
DFSV	domestic, family, and sexual violence
ED	emergency department
IPV	intimate partner violence
mHealth	mobile health
NGO	non-governmental organization
PCC	population–concept–context
PTSD	post-traumatic stress disorder
RCT	randomised controlled trial
SACC	sexual assault care centre
SANE	sexual assault nurse examiner
SARC	sexual assault referral centre
TVIC	trauma-and-violence-informed care

## References

[1] World Health Organization (2013). Responding to Intimate Partner Violence and Sexual Violence Against Women: WHO Clinical and Policy Guidelines.

[2] World Health Organization (2014). Health Care for Women Subjected to Intimate Partner Violence or Sexual Violence: A Clinical Handbook.

[3] Davies M., Satyen L., Toumbourou J.W. (2025). Trauma-and-violence-informed care for victim-survivors of domestic, family and sexual violence: A qualitative meta-synthesis of service providers’ perspectives. Trauma Violence Abus..

[4] Campbell R. (2024). Systems-centered care versus survivor-centered care: Reimagining help and healing for sexual assault survivors. Psychol. Violence.

[5] Satyen L., Sharda A., Robinson C. (2025). ‘You feel like an individual who matters’: The Beyond DV Recovery Pillars framework and its impact on the recovery and healing of victim-survivors of domestic, family and sexual violence. BMC Public Health.

[6] World Health Organization (2017). Strengthening Health Systems to Respond to Women Subjected to Intimate Partner Violence or Sexual Violence: A Manual for Health Managers.

[7] Substance Abuse and Mental Health Services Administration (2014). Trauma-Informed Care in Behavioral Health Services.

[8] Brooker C., Durmaz E. (2015). Mental health, sexual violence and the work of Sexual Assault Referral Centres (SARCs) in England. J. Forensic Leg. Med..

[9] Lewis-O’Connor A., Rittenberg E., Gerber M.R., Maitra A., Mitchell C. (2025). Trauma-informed care for intimate partner violence. Intimate Partner Violence: A Health-Based Perspective.

[10] National Institute for Health and Care Excellence (2014). Domestic Violence and Abuse: Multi-Agency Working.

[11] Kusmaul N., Wilson B., Nochajski T. (2015). The infusion of trauma-informed care in organizations: Experience of agency staff. Hum. Serv. Organ. Manag. Leadersh. Gov..

[12] Italia Decreto del Presidente del Consiglio dei Ministri 24 Novembre 2017. Linee Guida Nazionali Per le Aziende Sanitarie e le Aziende Ospedaliere in Tema di Soccorso e Assistenza Socio-Sanitaria Alle Donne Vittime di Violenza. Gazz. Uff. 2018, Serie Generale n. 24, 30 January 2018. https://www.gazzettaufficiale.it/eli/id/2018/01/30/18A00520/SG.

[13] Dipartimento per le Pari Opportunità (2025). Piano Strategico Nazionale Sulla Violenza Maschile Contro le Donne e la Violenza Domestica 2025–2027.

[14] Istituto Nazionale di Statistica (2026). I Centri Antiviolenza e le Donne Che Hanno Avviato il Percorso di Uscita Dalla Violenza—Anno 2024.

[15] Gabellini E., Salvatori A., Greco M.T., Cattaneo C., Tambuzzi S., Costantino M.A., Russo A.G. (2025). Access to health services by women subjected to violence: Findings from administrative healthcare data from the metropolitan area of northern Italy. BMC Women’s Health.

[16] De Paola L., Tripi D., Napoletano G., Marinelli E., Vergallo G.M., Zaami S. (2024). Violence against women within Italian and European context: Italian ‘Pink Code’—Major injuries and forensic expertise of a socio-cultural problem. Forensic Sci..

[17] Williams K., Harb M., Satyen L., Davies M. (2024). s-CAPE trauma recovery program: The need for a holistic, trauma- and violence-informed domestic violence framework. Front. Glob. Women’s Health.

[18] Darnell D., Peterson R., Berliner L., Stewart T., Russo J., Whiteside L., Zatzick D. (2015). Factors associated with follow-up attendance among rape victims seen in acute medical care. Psychiatry.

[19] Triandafilidis Z., Hobden B., Richardson S., Carey M. (2025). ‘They can build up trust again, so that health is not such a scary place’: The acceptability and feasibility of a multidisciplinary primary care outreach service for women affected by domestic, family and sexual violence. Aust. J. Prim. Health.

[20] Westwood T., Wendt S., Seymour K. (2020). Women’s perceptions of safety after domestic violence: Exploring experiences of a safety contact program. Affilia.

[21] Abrahams N., Gevers A. (2017). A rapid appraisal of the status of mental health support in post-rape care services in the Western Cape. S. Afr. J. Psychiatr..

[22] Baert S., Gilles C., Van Belle S., Bicanic I., Roelens K., Keygnaert I. (2021). Piloting sexual assault care centres in Belgium: Who do they reach and what care is offered?. Eur. J. Psychotraumatol..

[23] Bicanic I., Snetselaar H., De Jongh A., Van de Putte E. (2014). Victims’ use of professional services in a Dutch sexual assault centre. Eur. J. Psychotraumatol..

[24] Gupta J., Falb K.L., Ponta O., Xuan Z., Campos P.A., Gomez A.A., Valades J., Cariño G., Olavarrieta C.D. (2017). A nurse-delivered, clinic-based intervention to address intimate partner violence among low-income women in Mexico City: Findings from a cluster randomized controlled trial. BMC Med..

[25] Foa E.B., McLean C.P., Capaldi S., Rosenfield D. (2013). Prolonged exposure vs supportive counseling for sexual abuse-related PTSD in adolescent girls: A randomized clinical trial. JAMA.

[26] Nixon R.D.V., Best T., Wilksch S.R., Angelakis S., Beatty L.J., Weber N. (2016). Cognitive processing therapy for the treatment of acute stress disorder following sexual assault: A randomised effectiveness study. Behav. Change.

[27] Fagen J., Talarico R., Mercier O., Horth C., Souza S.C.S., Muldoon K.A., Sampsel K. (2025). Using telemedicine and virtual healthcare to improve clinical follow-up for survivors of sexual assault and intimate partner violence: A 7-year investigation of emergency department cases. Can. J. Emerg. Med..

[28] Ferrari G., Feder G., Agnew-Davies R., Bailey J.E., Hollinghurst S., Howard L., Howarth E., Sardinha L., Sharp D., Peters T.J. (2018). Psychological advocacy towards healing (PATH): A randomized controlled trial of a psychological intervention in a domestic violence service setting. PLoS ONE.

[29] Sullivan C.M., Simmons C., Guerrero M., Farero A., López-Zerón G., Ayeni O.O., Chiaramonte D., Sprecher M., Fernandez A.I. (2023). Domestic Violence Housing First Model and association with survivors’ housing stability, safety, and well-being over 2 years. JAMA Netw. Open.

[30] Hollingdrake O., Alban Cruz A., Currie J. (2025). A qualitative study exploring service users’ perspectives of the impact of a community-based nurse-led domestic violence service on women’s access to healthcare. BMC Nurs..

[31] Rizo C.F., Wretman C.J., Macy R.J., Guo S., Ermentrout D.M. (2018). A novel intervention for system-involved female intimate partner violence survivors: Changes in mental health. Am. J. Orthopsychiatry.

[32] Miller K.E., Cranston C.C., Davis J.L., Newman E., Resnick H. (2015). Psychological outcomes after a sexual assault video intervention: A randomized trial. J. Forensic Nurs..

[33] Walsh K., Gilmore A.K., Frazier P., Ledray L., Acierno R., Ruggiero K.J., Kilpatrick D.G., Resnick H.S. (2017). A randomized clinical trial examining the effect of video-based prevention of alcohol and marijuana use among recent sexual assault victims. Alcohol Clin. Exp. Res..

[34] Rajan G., Wachtler C., Lee S., Wändell P., Philips B., Wahlström L., Svedin C.G., Carlsson A.C. (2022). A one-session treatment of PTSD after single sexual assault trauma. A pilot study of the WONSA MLI Project: A randomized controlled trial. J. Interpers. Violence.

[35] Littleton H., Grills A.E., Kline K.D., Schoemann A.M., Dodd J.C. (2016). The From Survivor to Thriver Program: RCT of an online therapist-facilitated program for rape-related PTSD. J. Anxiety Disord..

[36] Hicks D.L., Patterson D., Resko S. (2017). Lessons learned from iCare: A postexamination text-messaging-based program with sexual assault patients. J. Forensic Nurs..

[37] Healey L.M., Hutchinson J.L., Pfeiffer M.N., Garton L., Hatten B., Dobbie M., Simpson L., Templeton D.J. (2023). The challenge of providing medical follow-up for sexual assault victims: Can we predict who will attend? A retrospective cross-sectional study. Sex. Health.

[38] Engleton J., Goodman-Williams R., Javorka M., Gregory K., Campbell R. (2022). Sexual assault survivors’ engagement with advocacy services during the COVID-19 pandemic. J. Community Psychol..

[39] Dworkin E.R., Schallert M., Lee C.M., Kaysen D. (2023). mHealth early intervention to reduce posttraumatic stress and alcohol use after sexual assault (THRIVE): Feasibility and acceptability results from a pilot trial. JMIR Form. Res..

[40] Mercier O., Parpia R., Presseau J., Muldoon K.A., Sampsel K. (2024). Telemedicine and virtual healthcare for survivors of sexual assault and intimate partner violence: A qualitative study. Women’s Health.

[41] Johnson D.M., Palmieri P.A., Zlotnick C., Hoffman L., Johnson N.L., Holmes S.C., Ceroni T.L. (2020). A randomized controlled trial comparing HOPE treatment and present-centered therapy in women residing in shelter with PTSD from IPV. Psychol. Women Q..

[42] Santos A., Matos M., Machado A. (2017). Effectiveness of a group intervention program for female victims of intimate partner violence. Small Group Res..

[43] Naismith I., Ripoll K., Pardo V.M. (2021). Group compassion-based therapy for female survivors of intimate-partner violence and gender-based violence: A pilot study. J. Fam. Violence.

[44] Li Y., Rhee H., Bullock L.F.C., McCaw B., Bloom T. (2024). Self-compassion, health, and empowerment: A pilot randomized controlled trial for Chinese immigrant women experiencing intimate partner violence. J. Interpers. Violence.

[45] Cheung D.S.T., Deng W., Tsao S.W., Ho R.T.H., Chan C.L.W., Fong D.Y.T., Chau P.H., Hong A.W.L., Fung H.Y.K.Y., Ma J.L.C. (2019). Effect of a Qigong intervention on telomerase activity and mental health in Chinese women survivors of intimate partner violence: A randomized clinical trial. JAMA Netw. Open.

[46] Foschiera L.N., de Freitas C.P.P., Luft C.Z., Godoi A.R., Dupont M.F., Habigzang L.F. (2024). Evidence of effectiveness of a psychotherapy protocol for women with a history of intimate partner violence: Follow-up study. Trends Psychol..

[47] Andersson G., Olsson E., Ringsgård E., Sandgren T., Viklund I., Andersson C., Hesselman Y., Johansson R., Nordgren L.B., Bohman B. (2021). Individually tailored Internet-delivered cognitive-behavioral therapy for survivors of intimate partner violence: A randomized controlled pilot trial. Internet Interv..

[48] Sabri B., Mata T., Li J., Butter S., Campbell J.C., Budhathoki C. (2024). The digital MySteps intervention for abused women at risk for firearm-related injuries and homicides: Findings from the feasibility, acceptability and preliminary efficacy trial. Contemp. Clin. Trials Commun..

[49] Sullivan C.M., López-Zerón G., Farero A., Ayeni O.O., Simmons C., Chiaramonte D., Guerrero M., Hamdan N., Sprecher M. (2023). Impact of the Domestic Violence Housing First Model on survivors’ safety and housing stability: Six month findings. J. Fam. Violence.

[50] Sullivan C.M., Guerrero M., Simmons C., López-Zerón G., Ayeni O.O., Farero A., Chiaramonte D., Sprecher M. (2023). Impact of the Domestic Violence Housing First Model on survivors’ safety and housing stability: 12-month findings. J. Interpers. Violence.

[51] Nnawulezi N., Macy R., Wretman C., Radtke S., Clark D.A., Sullivan C.M. (2025). Separate and cumulative impacts of trauma-informed practices and a housing intervention on the safety, housing stability and mental health of domestic violence survivors over two years. J. Fam. Violence.

[52] Rodgers M.A., Grisso J.A., Crits-Christoph P., Rhodes K.V. (2017). No quick fixes: A mixed methods feasibility study of an urban community health worker outreach program for intimate partner violence. Violence Against Women.

[53] Trevillion K., Byford S., Cary M., Rose D., Oram S., Feder G., Agnew-Davies R., Howard L.M. (2014). Linking abuse and recovery through advocacy: An observational study. Epidemiol. Psychiatr. Sci..

[54] Brignone L., Gomez A.M. (2022). Access to domestic violence advocacy by race, ethnicity and gender: The impact of a digital warm handoff from the emergency department. PLoS ONE.

[55] Halliwell G., Dheensa S., Fenu E., Jones S.K., Asato J., Jacob S., Feder G. (2019). Cry for health: A quantitative evaluation of a hospital-based advocacy intervention for domestic violence and abuse. BMC Health Serv. Res..

[56] Dheensa S., Halliwell G., Daw J., Jones S.K., Feder G. (2020). ‘From taboo to routine’: A qualitative evaluation of a hospital-based advocacy intervention for domestic violence and abuse. BMC Health Serv. Res..

[57] Berry O.O., Kaufman P., Weiss M., Fitelson E., Monk C. (2024). Co-location of specialized mental health services in an intimate partner violence advocacy organization. Med. Sci. Law.

[58] Hackenberg E.A.M., Sallinen V., Handolin L., Koljonen V. (2021). Victims of severe intimate partner violence are left without advocacy intervention in primary care emergency rooms: A prospective observational study. J. Interpers. Violence.

[59] Sorrentino A.E., Iverson K.M., Tuepker A., True G., Cusack M., Newell S., Dichter M.E. (2021). Mental health care in the context of intimate partner violence: Survivor perspectives. Psychol. Serv..

[60] van Gelder N.E., Ligthart S.A., van Rosmalen-Nooijens K.A.W.L., Prins J.B., Oertelt-Prigione S. (2023). Effectiveness of the SAFE eHealth intervention for women experiencing intimate partner violence and abuse: Randomized controlled trial, quantitative process evaluation, and open feasibility study. J. Med. Internet Res..

[61] Rizo C.F., Reynolds A., Macy R.J., Ermentrout D.M. (2016). Parenting and Safety Program for system-involved female survivors of intimate partner violence: A qualitative follow-up study. J. Fam. Violence.

[62] NHS England (2018). National Service Specification for Sexual Assault Referral Centres.

[63] Sitoh A., Whitehouse C. (2026). Care in the aftermath: A scoping review of acute sexual assault response models and their documented effects. Trauma Violence Abus.

[64] Sanhueza-Morales T., Michaelsen S., Touati N., del Barrio L.R. (2025). Barriers in accessing intimate partner violence services: Intersecting views of immigrant and minority ethnic survivors and community organization workers. Women’s Health.

[65] Macy R.J., Martin S.L., Ogbonnaya I.N., Rizo C.F. (2018). What do domestic violence and sexual assault service providers need to know about survivors to deliver services?. Violence Against Women.

[66] Dupuis A., Thorns D.C. (1998). Home, home ownership and the search for ontological security. Sociol. Rev..

[67] Padgett D.K. (2007). There’s no place like (a) home: Ontological security among persons with serious mental illness in the United States. Soc. Sci. Med..

[68] Storer H.L., Nyerges E.X. (2023). The rapid uptake of digital technologies at domestic violence and sexual assault organizations during the COVID-19 pandemic. Violence Against Women.

[69] Bach M.H., Krogh S.N.S., Hansen M. (2025). ‘That kind of information is crucial to get across’: Co-developing a sexual assault support website with survivors and support providers. Int. J. Qual. Stud. Health Well-Being.

[70] Yu P., Zhu P., Kudiza A., Kaburu F.M., Intizar M., Tong C., Zhang R., Jiang J., Yu X., Kuang Q. (2026). Help, near and far: A systematic review of post-COVID digital mental health solutions for domestic violence victims. Front. Public Health.

[71] Iraola E., Menard J.-P., Baranne M.-L., Cudonnec J., Buresi I., Chariot P. (2024). Low uptake of gynecological consultation following domestic or sexual violence: A case-control study during pregnancy follow-up. Eur. J. Obstet. Gynecol. Reprod. Biol..

[72] Muftić L.R., Hoppe S., Grubb J.A. (2019). The use of help seeking and coping strategies among Bosnian women in domestic violence shelters. J. Gend.-Based Violence.

[73] Li Y., Dong F., Bullock L.F.C., Bloom T. (2022). Exploring help-seeking experiences of Chinese immigrant survivors of intimate partner violence in the United States. Psychol. Trauma.

[74] Pallansch J., Milam C., Ham K., Morgan P., Manning J., Salzman J., Kopec K., Lewis M. (2022). Intimate partner violence, sexual assault, and child abuse resource utilization during COVID-19. West. J. Emerg. Med..

[75] Catherine N.L.A., MacMillan H., Jack S., Zheng Y., Xie H., Boyle M., Sheehan D., Gonzalez A., Gafni A., Tonmyr L. (2025). Effects of nurse-home visiting on intimate partner violence and maternal income, mental health and self-efficacy by 24 months postpartum: A randomised controlled trial (British Columbia Healthy Connections Project). BMJ Open.

