# Supplementary material for: Post-Acute Care Pathways After Sexual Violence and Intimate Partner Violence: An International Health-Services Scoping Review with Implications for Italy
Source: Healthcare (Basel). 2026 Jun 16;14(12):1735. doi: 10.3390/healthcare14121735 (PMC13300668; doi:10.3390/healthcare14121735)
Supplement: Supplementary file 1 [file healthcare-14-01735-s001.zip › healthcare-4319728-supplementary.pdf]

Supplementary Materials

Post-acute care pathways after sexual violence and intimate partner violence

Supplementary Files S1-S4. Search strategies, PRISMA-ScR checklist, data charting form and populated extraction matrix with post-acute entry points/follow-up horizons, and full-text exclusions

Supplementary File S1. Full search strategy

Search date for all databases: final revised searches conducted in April 2026. Limits: English language; publication year 2013 onward. For Web of Science, year and language limits were applied through platform filters. For Embase.com, APA PsycINFO on EBSCOhost, and CINAHL on EBSCOhost, language/year limits were applied within the platform. Table S1 reports the number of records retrieved by database and query stream.

Table S1. Records retrieved by database and query stream.

Theme	Query stream	Database	Records
Domestic violence / IPV	CORE	Embase	581
Domestic violence / IPV	CORE	APA PsycINFO	441
Domestic violence / IPV	CORE	PubMed	1246
Domestic violence / IPV	CORE	Scopus	825
Domestic violence / IPV	CORE	WoS	690
Domestic violence / IPV	CORE	CINAHL	326
Domestic violence / IPV	MENTAL HEALTH SUPPLEMENT	Embase	69
Domestic violence / IPV	MENTAL HEALTH SUPPLEMENT	APA PsycINFO	36
Domestic violence / IPV	MENTAL HEALTH SUPPLEMENT	PubMed	380
Domestic violence / IPV	MENTAL HEALTH SUPPLEMENT	Scopus	123
Domestic violence / IPV	MENTAL HEALTH SUPPLEMENT	WoS	80
Domestic violence / IPV	MENTAL HEALTH SUPPLEMENT	CINAHL	34

Sexual violence / sexual assault	CORE	Embase	731
Sexual violence / sexual assault	CORE	APA PsycINFO	531
Sexual violence / sexual assault	CORE	PubMed	1110
Sexual violence / sexual assault	CORE	Scopus	890
Sexual violence / sexual assault	CORE	WoS	736
Sexual violence / sexual assault	CORE	CINAHL	318
Sexual violence / sexual assault	MENTAL HEALTH SUPPLEMENT	Embase	68
Sexual violence / sexual assault	MENTAL HEALTH SUPPLEMENT	APA PsycINFO	36
Sexual violence / sexual assault	MENTAL HEALTH SUPPLEMENT	PubMed	366
Sexual violence / sexual assault	MENTAL HEALTH SUPPLEMENT	Scopus	95
Sexual violence / sexual assault	MENTAL HEALTH SUPPLEMENT	WoS	54
Sexual violence / sexual assault	MENTAL HEALTH SUPPLEMENT	CINAHL	22

PubMed

Sexual violence / sexual assault — CORE

```
 (
(
  "Rape"[majr] OR "Sex Offenses"[majr]
  OR rape[ti]
  OR "sexual assault"[ti]
  OR "sexual violence"[ti]
)
AND
(
  "follow-up"[tiab]
  OR "follow up"[tiab]
  OR aftercare[tiab]
  OR "post-assault"[tiab]
  OR postassault[tiab]
  OR "post-acute"[tiab]
  OR postacute[tiab]
  OR "continuity of care"[tiab]
  OR "care pathway"[tiab]
  OR "care pathways"[tiab]
  OR advocacy[tiab]
  OR "case management"[tiab]
  OR "care coordination"[tiab]
```

```
    OR telehealth[tiab]
    OR telemedicine[tiab]
    OR mhealth[tiab]
  )
)
AND ("2013/01/01"[dp] : "3000"[dp])
AND english[lang]
```

Domestic violence / IPV — CORE

```
(
  (
    "Domestic Violence"[majr]
    OR "domestic violence"[ti]
    OR "intimate partner violence"[ti]
    OR "partner violence"[ti]
  )
  AND
  (
    "follow-up"[tiab]
    OR "follow up"[tiab]
    OR aftercare[tiab]
    OR "post-crisis"[tiab]
    OR "post-acute"[tiab]
    OR postacute[tiab]
    OR "continuity of care"[tiab]
    OR "care pathway"[tiab]
    OR "care pathways"[tiab]
    OR "safety planning"[tiab]
  )
)
AND ("2013/01/01"[dp] : "3000"[dp])
AND english[lang]
```

Sexual violence / sexual assault — MENTAL HEALTH SUPPLEMENT

```
(
  (
    "Rape"[Mesh]
    OR "Sex Offenses"[Mesh]
    OR rape[tiab]
    OR "sexual assault"[tiab]
    OR "sexual violence"[tiab]
    OR "sexual abuse"[tiab]
  )
  AND
  (
    psychotherapy[tiab]
    OR counselling[tiab]
    OR counseling[tiab]
    OR "mental health support"[tiab]
    OR "psychological support"[tiab]
    OR "psychological care"[tiab]
    OR "mental health care"[tiab]
    OR PTSD[tiab]
    OR "posttraumatic stress"[tiab]
    OR trauma-focused[tiab]
  )
  AND
  (
    "follow-up"[tiab]
```

```
    OR "follow up"[tiab]
    OR aftercare[tiab]
    OR "post-assault"[tiab]
    OR postassault[tiab]
    OR "post-acute"[tiab]
    OR postacute[tiab]
    OR "continuity of care"[tiab]
    OR "care pathway"[tiab]
    OR "care pathways"[tiab]
    OR "case management"[tiab]
    OR "care coordination"[tiab]
  )
)
AND ("2013/01/01"[dp] : "3000"[dp])
AND english[lang]
```

Domestic violence / IPV — MENTAL HEALTH SUPPLEMENT

```
(
  (
    "Domestic Violence"[Mesh]
    OR "domestic violence"[tiab]
    OR "intimate partner violence"[tiab]
    OR IPV[tiab]
    OR "partner violence"[tiab]
    OR "gender-based violence"[tiab]
  )
  AND
  (
    psychotherapy[tiab]
    OR counselling[tiab]
    OR counseling[tiab]
    OR "mental health support"[tiab]
    OR "psychological support"[tiab]
    OR "psychological care"[tiab]
    OR "mental health care"[tiab]
    OR PTSD[tiab]
    OR "posttraumatic stress"[tiab]
    OR trauma-focused[tiab]
  )
  AND
  (
    "follow-up"[tiab]
    OR "follow up"[tiab]
    OR aftercare[tiab]
    OR "post-crisis"[tiab]
    OR "post-acute"[tiab]
    OR postacute[tiab]
    OR "continuity of care"[tiab]
    OR "care pathway"[tiab]
    OR "care pathways"[tiab]
    OR "case management"[tiab]
    OR "care coordination"[tiab]
    OR "safety planning"[tiab]
  )
)
AND ("2013/01/01"[dp] : "3000"[dp])
AND english[lang]
```

Scopus

Sexual violence / sexual assault — CORE

```
  (
TITLE(rape OR "sexual assault" OR "sexual violence")
AND
TITLE-ABS-KEY(
  "follow-up" OR "follow up" OR aftercare
  OR "post-assault" OR postassault
  OR "post-acute" OR postacute
  OR "continuity of care"
  OR "care pathway" OR "care pathways"
  OR advocacy
  OR "case management"
  OR "care coordination"
  OR telehealth OR telemedicine OR mhealth
)
)
AND PUBYEAR > 2012
AND (LIMIT-TO(LANGUAGE, "English"))
```

Domestic violence / IPV — CORE

```
  (
TITLE("domestic violence" OR "intimate partner violence" OR "partner violence")
AND
TITLE-ABS-KEY(
  "follow-up" OR "follow up" OR aftercare
  OR "post-crisis"
  OR "post-acute" OR postacute
  OR "continuity of care"
  OR "care pathway" OR "care pathways"
  OR "safety planning"
)
)
AND PUBYEAR > 2012
AND (LIMIT-TO(LANGUAGE, "English"))
```

Sexual violence / sexual assault — MENTAL HEALTH SUPPLEMENT

```
  (
TITLE("sexual assault" OR "sexual violence" OR rape)
AND
TITLE-ABS-KEY(
  psychotherapy OR counseling OR counselling
  OR "mental health support"
  OR "psychological support"
  OR "psychological care"
  OR "mental health care"
  OR trauma-focused
)
AND
TITLE-ABS-KEY(
  "follow-up" OR "follow up" OR aftercare
  OR "post-assault" OR postassault
  OR "post-acute" OR postacute
  OR "continuity of care"
  OR "care pathway" OR "care pathways"
)
)
```

```
AND PUBYEAR > 2012
AND (LIMIT-TO(LANGUAGE, "English"))
```

Domestic violence / IPV — MENTAL HEALTH SUPPLEMENT

```
(
  TITLE("domestic violence" OR "intimate partner violence" OR "partner violence")
  AND
  TITLE-ABS-KEY(
    psychotherapy OR counseling OR counselling
    OR "mental health support"
    OR "psychological support"
    OR "psychological care"
    OR "mental health care"
    OR trauma-focused
  )
  AND
  TITLE-ABS-KEY(
    "follow-up" OR "follow up" OR aftercare
    OR "post-crisis"
    OR "post-acute" OR postacute
    OR "continuity of care"
    OR "care pathway" OR "care pathways"
    OR "safety planning"
  )
)
AND PUBYEAR > 2012
AND (LIMIT-TO(LANGUAGE, "English"))
```

Web of Science Core Collection

Sexual violence / sexual assault — CORE

```
(
  TI=(rape OR "sexual assault" OR "sexual violence")
  AND
  TS=("follow-up" OR "follow up" OR aftercare
      OR "post-assault" OR postassault
      OR "post-acute" OR postacute
      OR "continuity of care"
      OR "care pathway" OR "care pathways"
      OR advocacy
      OR "case management"
      OR "care coordination"
      OR telehealth OR telemedicine OR mhealth)
)
AND LA=(English)
AND PY=(2013-2026)
```

Domestic violence / IPV — CORE

```
(
  TI=("domestic violence" OR "intimate partner violence" OR "partner violence")
  AND
  TS=("follow-up" OR "follow up" OR aftercare
      OR "post-crisis"
      OR "post-acute" OR postacute
      OR "continuity of care"
      OR "care pathway" OR "care pathways"
      OR "safety planning")
)
```

```
AND LA=(English)
AND PY=(2013-2026)
```

Sexual violence / sexual assault — MENTAL HEALTH SUPPLEMENT

```
(
  TI=("sexual assault" OR "sexual violence" OR rape)
  AND
  TS=(psychotherapy OR counseling OR counselling
      OR "mental health support"
      OR "psychological support"
      OR "psychological care"
      OR "mental health care"
      OR trauma-focused)
  AND
  TS=("follow-up" OR "follow up" OR aftercare
      OR "post-assault" OR postassault
      OR "post-acute" OR postacute
      OR "continuity of care"
      OR "care pathway" OR "care pathways")
)
AND LA=(English)
AND PY=(2013-2026)
```

Domestic violence / IPV — MENTAL HEALTH SUPPLEMENT

```
(
  TI=("domestic violence" OR "intimate partner violence" OR "partner violence")
  AND
  TS=(psychotherapy OR counseling OR counselling
      OR "mental health support"
      OR "psychological support"
      OR "psychological care"
      OR "mental health care"
      OR trauma-focused)
  AND
  TS=("follow-up" OR "follow up" OR aftercare
      OR "post-crisis"
      OR "post-acute" OR postacute
      OR "continuity of care"
      OR "care pathway" OR "care pathways"
      OR "safety planning")
)
AND LA=(English)
AND PY=(2013-2026)
```

Embase.com

Sexual violence / sexual assault — CORE

```
(rape:ti OR 'sexual assault':ti OR 'sexual violence':ti) AND ('follow-up':ti,ab,kw OR 'follow up':ti,ab,kw OR aftercare:ti,ab,kw OR 'post-assault':ti,ab,kw OR postassault:ti,ab,kw OR 'post-acute':ti,ab,kw OR postacute:ti,ab,kw OR 'continuity of care':ti,ab,kw OR 'care pathway':ti,ab,kw OR 'care pathways':ti,ab,kw OR advocacy:ti,ab,kw OR 'case management':ti,ab,kw OR 'care coordination':ti,ab,kw OR telehealth:ti,ab,kw OR telemedicine:ti,ab,kw OR mhealth:ti,ab,kw)
```

Domestic violence / IPV — CORE

```
('domestic violence':ti OR 'intimate partner violence':ti OR 'partner violence':ti) AND ('follow-up':ti,ab,kw OR 'follow up':ti,ab,kw OR aftercare:ti,ab,kw OR 'post-crisis':ti,ab,kw OR 'post-acute':ti,ab,kw OR postacute:ti,ab,kw OR 'continuity of care':ti,ab,kw OR 'care pathway':ti,ab,kw OR 'care pathways':ti,ab,kw OR 'safety planning':ti,ab,kw)
```

Sexual violence / sexual assault — MENTAL HEALTH SUPPLEMENT

```
('sexual assault':ti OR 'sexual violence':ti OR rape:ti) AND (psychotherapy:ti,ab,kw OR counseling:ti,ab,kw OR counselling:ti,ab,kw OR 'mental health support':ti,ab,kw OR 'psychological support':ti,ab,kw OR 'psychological care':ti,ab,kw OR 'mental health care':ti,ab,kw OR 'trauma focused':ti,ab,kw) AND ('follow-up':ti,ab,kw OR 'follow up':ti,ab,kw OR aftercare:ti,ab,kw OR 'post-assault':ti,ab,kw OR postassault:ti,ab,kw OR 'post-acute':ti,ab,kw OR postacute:ti,ab,kw OR 'continuity of care':ti,ab,kw OR 'care pathway':ti,ab,kw OR 'care pathways':ti,ab,kw)
```

Domestic violence / IPV — MENTAL HEALTH SUPPLEMENT

```
('domestic violence':ti OR 'intimate partner violence':ti OR 'partner violence':ti) AND (psychotherapy:ti,ab,kw OR counseling:ti,ab,kw OR counselling:ti,ab,kw OR 'mental health support':ti,ab,kw OR 'psychological support':ti,ab,kw OR 'psychological care':ti,ab,kw OR 'mental health care':ti,ab,kw OR 'trauma focused':ti,ab,kw) AND ('follow-up':ti,ab,kw OR 'follow up':ti,ab,kw OR aftercare:ti,ab,kw OR 'post-crisis':ti,ab,kw OR 'post-acute':ti,ab,kw OR postacute:ti,ab,kw OR 'continuity of care':ti,ab,kw OR 'care pathway':ti,ab,kw OR 'care pathways':ti,ab,kw OR 'safety planning':ti,ab,kw)
```

APA PsycINFO via EBSCOhost

Sexual violence / sexual assault — CORE

TI (rape OR "sexual assault" OR "sexual violence") AND AB ("follow-up" OR "follow up" OR aftercare OR "post-assault" OR postassault OR "post-acute" OR postacute OR "continuity of care" OR "care pathway" OR "care pathways" OR advocacy OR "case management" OR "care coordination" OR telehealth OR telemedicine OR mhealth)

Domestic violence / IPV — CORE

TI ("domestic violence" OR "intimate partner violence" OR "partner violence") AND AB ("follow-up" OR "follow up" OR aftercare OR "post-crisis" OR "post-acute" OR postacute OR "continuity of care" OR "care pathway" OR "care pathways" OR "safety planning")

Sexual violence / sexual assault — MENTAL HEALTH SUPPLEMENT

TI ("sexual assault" OR "sexual violence" OR rape) AND AB (psychotherapy OR counseling OR counselling OR "mental health support" OR "psychological support" OR "psychological care" OR "mental health care" OR "trauma focused") AND AB ("follow-up" OR "follow up" OR aftercare OR "post-assault" OR postassault OR "post-acute" OR postacute OR "continuity of care" OR "care pathway" OR "care pathways")

Domestic violence / IPV — MENTAL HEALTH SUPPLEMENT

TI ("domestic violence" OR "intimate partner violence" OR "partner violence") AND AB (psychotherapy OR counseling OR counselling OR "mental health support" OR "psychological support" OR "psychological care" OR "mental health care" OR "trauma focused") AND AB ("follow-up" OR "follow up" OR aftercare OR "post-crisis" OR "post-acute" OR postacute OR "continuity of care" OR "care pathway" OR "care pathways" OR "safety planning")

CINAHL via EBSCOhost

Sexual violence / sexual assault — CORE

TI (rape OR "sexual assault" OR "sexual violence") AND AB ("follow-up" OR "follow up" OR aftercare OR "post-assault" OR postassault OR "post-acute" OR postacute OR "continuity of care" OR "care pathway" OR "care pathways" OR advocacy OR "case management" OR "care coordination" OR telehealth OR telemedicine OR mhealth)

Domestic violence / IPV — CORE

TI ("domestic violence" OR "intimate partner violence" OR "partner violence") AND AB ("follow-up" OR "follow up" OR aftercare OR "post-crisis" OR "post-acute" OR postacute OR "continuity of care" OR "care pathway" OR "care pathways" OR "safety planning")

Sexual violence / sexual assault — MENTAL HEALTH SUPPLEMENT

TI ("sexual assault" OR "sexual violence" OR rape) AND AB (psychotherapy OR counseling OR counselling OR "mental health support" OR "psychological support" OR "psychological care" OR "mental health care" OR "trauma focused") AND AB ("follow-up" OR "follow up" OR aftercare OR "post-assault" OR postassault OR "post-acute" OR postacute OR "continuity of care" OR "care pathway" OR "care pathways")

Domestic violence / IPV — MENTAL HEALTH SUPPLEMENT

TI ("domestic violence" OR "intimate partner violence" OR "partner violence") AND AB (psychotherapy OR counseling OR counselling OR "mental health support" OR "psychological support" OR "psychological care" OR "mental health care" OR "trauma focused") AND AB ("follow-up" OR "follow up" OR aftercare OR "post-crisis" OR "post-acute" OR postacute OR "continuity of care" OR "care pathway" OR "care pathways" OR "safety planning")

Search and screening summary

The final revised six-database search architecture yielded 9,788 records before deduplication, 3,944 records after within-theme deterministic deduplication, and 3,708 unique records after cross-theme consolidation. Title/abstract screening of the final revised corpus led to 84 reports being sought for retrieval; therefore, 3,624 records were excluded at title/abstract screening. Aggregate reasons for title/abstract exclusion were: not meeting Population-Concept-Context criteria; acute/emergency/forensic or first-contact focus only; screening, hotline, referral-only, awareness, or service-access studies without an identifiable post-acute continuity mechanism; wrong publication type; or irrelevant population, setting, or concept. Three reports were not retrieved and 81 reports were assessed in full text. Forty-four studies met the inclusion criteria for the charted core synthesis. 37 reports were not included in the core synthesis after full-text assessment because they were acute/first-contact studies, screening or referral-only models, conference abstracts, reviews, protocols, adjacent papers without a sufficiently identifiable post-acute pathway, or otherwise outside the operational eligibility boundary.

Locations are reported by manuscript section or manuscript element rather than final pagination, because journal-specific formatting may alter page numbering at submission stage.

Supplementary File S2

Table S2. PRISMA-ScR reporting checklist with manuscript locations.

Section / topic	Item	PRISMA-ScR checklist item	Location in manuscript
TITLE — Title	1	Identify the report as a scoping review.	Title
ABSTRACT — Structured summary	2	Provide a structured summary that includes (as applicable): background, objectives, eligibility criteria, sources of evidence, charting methods, results, and conclusions that relate to the review questions and objectives.	Abstract

INTRODUCTION — Rationale	3	Describe the rationale for the review in the context of what is already known. Explain why the review questions/objectives lend themselves to a scoping review approach.	Introduction
INTRODUCTION — Objectives	4	Provide an explicit statement of the questions and objectives being addressed with reference to their key elements (e.g., population or participants, concepts, and context) or other relevant key elements used to conceptualize the review questions and/or objectives.	Introduction; Methods — review questions
METHODS — Protocol and registration	5	Indicate whether a review protocol exists; state if and where it can be accessed (e.g., a Web address); and if available, provide registration information, including the registration number.	Methods — no external protocol registered; internal review plan described
METHODS — Eligibility criteria	6	Specify characteristics of the sources of evidence (e.g., years considered, language, and publication status) used as criteria for eligibility, and provide a rationale.	Abstract; Methods — eligibility criteria
METHODS — Information sources	7	Describe all information sources in the search (e.g., databases with dates of coverage, contact with authors to identify additional sources), as well as the date the most recent search was executed.	Methods — information sources and search strategy; Supplementary materials statement; Supplementary File 1
METHODS — Search	8	Present the full electronic search strategy for at least 1 database, including any limits used, such that it could be repeated.	Methods — information sources and search strategy; Supplementary File 1
METHODS — Selection of sources of evidence	9	State the process for selecting sources of evidence (i.e., screening and eligibility) included in the scoping review.	Methods — study selection
METHODS — Data charting process	10	Describe the methods of charting data from the included sources of evidence (e.g., calibrated forms or forms tested by the team before their use, and whether data charting was done independently or in duplicate) and any processes for obtaining and confirming data from investigators.	Methods — data charting; Supplementary File 3
METHODS — Data items	11	List and define all variables for which data were sought and any assumptions and simplifications made.	Methods — data charting; Supplementary File 3; Supplementary Tables S3–S4
METHODS — Critical appraisal of individual sources of evidence (optional)	12	If done, provide a rationale for conducting a critical appraisal of included sources of evidence; describe the methods used and how this information was used in any data synthesis.	Not applicable — no formal critical appraisal performed
METHODS — Synthesis of results	13	Describe the methods of handling and summarizing the data that were charted.	Methods — data synthesis
RESULTS — Selection of sources of evidence	14	Give numbers of sources of evidence screened, assessed for eligibility, and included in the review, with reasons for exclusions at each stage, ideally using a flow diagram.	Methods — study selection; Figure 1; Results 3.1; Supplementary File 4
RESULTS — Characteristics of sources of evidence	15	For each source of evidence, present characteristics for which data were charted and provide the citations.	Results 3.2; Table 1; Supplementary Table S4

RESULTS — Critical appraisal within sources of evidence (optional)	16	If done, present data on critical appraisal of included sources of evidence.	Not applicable — no formal critical appraisal performed
RESULTS — Results of individual sources of evidence	17	For each included source of evidence, present the relevant data that were charted that relate to the review questions and objectives.	Results 3.3–3.7; Table 1; Supplementary Table S4
RESULTS — Synthesis of results	18	Summarize and/or present the charting results as they relate to the review questions and objectives.	Results 3.3–3.7; Tables 1–2
DISCUSSION — Summary of evidence	19	Summarize the main results, including an overview of concepts, themes, and types of evidence available; link to the review questions and objectives, and consider the relevance to key groups.	Discussion
DISCUSSION — Limitations	20	Discuss the limitations of the scoping review process.	Discussion — strengths and limitations
DISCUSSION — Conclusions	21	Provide a general interpretation of the results with respect to the review questions and objectives, as well as potential implications and/or next steps.	Discussion; Box 1; Conclusions
FUNDING — Funding	22	Describe sources of funding for the included sources of evidence, as well as sources of funding for the scoping review. Describe the role of the funders of the scoping review.	Funding statement (review funding); funding of individual included sources not clearly reported

Supplementary File S3. Data charting form and populated extraction matrix

The following variables were used during data charting and synthesis. The form was designed to support both descriptive mapping and cross-country organisational comparison. The populated matrix in Supplementary Table S4 reports the principal charted data for the 44 core studies.

Table S3. Data charting variables and definitions.

Domain	Variable	Operational note
Identification	Study ID	Short identifier used during extraction and synthesis.
Identification	Full citation	Author(s), year, title, journal/source, and DOI if available.
Context	Country	Country or countries in which the intervention/service was implemented.
Context	Setting	Hospital, sexual assault centre, shelter, community service, clinic, NGO, digital platform, or hybrid model.
Study characteristics	Study design	RCT, quasi-experimental, qualitative, mixed-methods, service evaluation, observational study, or other.
Population	Target population	Sexual assault survivors, domestic violence/IPV survivors, or mixed population.

Population	Age group	Adolescent, adult, or mixed.
Violence type	Type of violence	Sexual assault, rape, domestic violence, IPV, or mixed violence exposure.
Entry point	Initial service contact	Emergency department, forensic service, anti-violence centre, shelter, clinic, court/CPS referral, or community referral.
Post-acute definition	Timing/operational definition	How the study defined or implied the post-acute phase.
Intervention	Model/intervention name	Primary organisational or therapeutic model described.
Intervention	Core components	Clinical follow-up, psychotherapy, advocacy, case management, housing support, digital support, and other relevant elements.
Intervention	Duration/intensity	Number of sessions, follow-up horizon, or frequency/intensity of contact.
Workforce	Professionals involved	Nurses, physicians, psychologists, advocates, social workers, peer supporters, case managers, multidisciplinary teams.
Continuity	Mechanisms of continuity	Scheduled follow-up, warm handoff, telehealth, outreach, co-location, coordinated referral, or blended model.
Outcomes	Reported outcomes	Clinical, psychological, service-use, safety, housing, empowerment, wellbeing, or survivor-defined outcomes.
Implementation	Barriers/facilitators	Reported organisational, systemic, cultural, logistical, and survivor-level barriers or facilitators.
Interpretive field	Relevance for Italy	Brief note on the model's potential relevance for the Italian context.

Table S4. Populated extraction matrix of included core studies, including post-acute entry point, operational timing, continuity mechanism, and follow-up horizon.

Note. This table is intended to show the heterogeneity of post-acute entry points and follow-up horizons across the charted core studies. The post-acute boundary was therefore operationalised through the presence of an identifiable continuity mechanism beyond first contact rather than through a uniform time cut-off.

Study	Country	Violence type	Population / setting	Post-acute component	Continuity mechanism / workforce	Follow-up / outcomes	Synthesis relevance
Darnell et al. (2015) [18]	USA	Sexual violence	Rape victims seen in acute medical care after assault; Emergency/acute medical care; SANE-linked follow-	Scheduled medical/counselling follow-up after acute rape care	Scheduled follow-up appointment and linkage to medical/mental health services	Recommended post-assault follow-up; attendance assessed after acute visit; Follow-up attendance; predictors of	Central evidence on retention, dropout, and targeted linkage after

Study	Country	Violence type	Population / setting	Post-acute component	Continuity mechanism / workforce	Follow-up / outcomes	Synthesis relevance
			up			non-attendance; only a minority attended the recommended appointment	first access
Triandafilidis et al. (2025) [19]	Australia	Mixed DFSV	Women and children affected by domestic, family, and sexual violence; Multidisciplinary primary-care outreach service	Proactive, trauma-informed primary-care outreach with advocacy and multidisciplinary care	Flexible outreach, trusted provider contact, multidisciplinary communication, and facilitated linkage	Feasibility/acceptability assessment during service pilot; Acceptability and feasibility; trust-building; reduced access barriers; provider collaboration	Supports outreach-based, territorially embedded post-acute follow-up models
Westwood et al. (2019) [20]	Australia	IPV / domestic	Women whose partners/ex-partners were referred to a perpetrator intervention programme; Integrated domestic and family violence programme; women's safety service	Safety-contact programme linked to integrated perpetrator intervention	Telephone safety contact, emotional support, practical safety planning, and survivor-centred monitoring	Approximately 10 weeks of safety-contact support; Women's perceived safety, emotional support, trauma-focused practice, and recovery-oriented safety planning	Supports safety-contact follow-up and survivor-defined safety outcomes
Abrahams et al. (2017) [21]	South Africa	Sexual violence	Rape survivors using post-rape services; Post-rape care services	Integrated mental health support within post-rape services	Centre-based linkage, psychosocial assessment, or coordinated referral	Cross-sectional service snapshot; Availability of psychosocial/mental health support	Mental health integration into post-rape pathways
Baert et al. (2021) [22]	Belgium	Sexual violence	Victims using SACCs; Sexual Assault Care Centres	Integrated multidisciplinary sexual assault centre model	Centre-based linkage, psychosocial assessment, or coordinated referral	Service pathway description; Reach, care components offered	Transferable integrated pathway model
Bicanic et al. (2014) [23]	Netherlands	Sexual violence	Victims attending sexual assault centre; Sexual assault centre	Integrated centre with professional follow-up/referral	Centre-based linkage, psychosocial assessment, or coordinated referral	Service utilization after assault; Use of medical, psychological and legal services	Actual use of post-assault services
Gupta et al. (2017) [24]	Mexico	IPV/domestic	Low-income women in Mexico City experiencing IPV; Clinic-based care	Nurse-delivered clinic intervention	Embedded healthcare contact and referral/support pathway	Trial follow-up; Safety/help-seeking/related outcomes	Clinic-based IPV response beyond initial disclosure
Foa et al. (2013) [25]	USA	Sexual violence	Adolescent girls with sexual abuse-related PTSD; Therapy / outpatient mental health	Trauma-focused psychotherapy	Scheduled therapeutic sessions with post-treatment assessment	Post-treatment follow-up; PTSD symptoms and related mental health outcomes	Specialized trauma therapy after sexual violence
Nixon et al. (2016) [26]	Australia	Sexual violence	Sexual assault survivors with acute stress disorder; Psychotherapy service	Early cognitive processing therapy	Scheduled therapeutic sessions with post-treatment assessment	Short-term follow-up; Acute stress / PTSD symptoms	Early post-assault psychotherapy
Fagen et al. (2025) [27]	Canada	Sexual violence	Survivors of sexual assault and IPV after ED care; Emergency department linked virtual follow-up	Telemedicine-enabled clinical follow-up	Remote contact, digital support, or telehealth-enabled continuity	7-year service investigation; Clinical follow-up uptake and service delivery	Virtual bridge from acute care to follow-up
Ferrari et al. (2018) [28]	United Kingdom	IPV/domestic	Women survivors of domestic violence; Domestic violence service setting	Psychological advocacy	Advocacy/case-management linkage and service navigation	Post-intervention follow-up; Psychological distress / mental health	Advocacy integrated with mental health support
Sullivan et al. (2023)	USA	IPV/domestic	Domestic violence survivors;	Housing First + survivor-	Longitudinal housing support and	24 months; Housing stability, safety,	Strong social recovery

Study	Country	Violence type	Population / setting	Post-acute component	Continuity mechanism / workforce	Follow-up / outcomes	Synthesis relevance
[29]			Housing + advocacy services	centered advocacy	stabilisation contacts	well-being	model beyond acute care
Hollingdrake et al. (2025) [30]	Australia	IPV/domestic	Women using domestic violence service; Community-based nurse-led service	Nurse-led domestic violence service	Embedded healthcare contact and referral/support pathway	Service-use perspectives; Access to healthcare and service experiences	Nurse-led linkage between IPV support and healthcare
Rizo et al. (2018) [31]	USA	IPV/domestic	Court/CPS-involved female IPV survivors; Community-based group intervention	13-week psychoeducational safety-parenting-mental health program	Scheduled therapeutic sessions with post-treatment assessment	3 and 6 months; Depression and PTSD symptoms	Mandated-service group with tangible follow-up outcomes
Miller et al. (2015) [32]	USA	Sexual violence	Recent sexual assault survivors; Post-assault care / forensic nursing context	Brief psychoeducational video intervention	Structured follow-up or service-use pathway	Short-term follow-up; Psychological symptoms / coping	Low-intensity standardized post-assault support
Walsh et al. (2017) [33]	USA	Sexual violence	Recent sexual assault victims; Post-assault intervention	Video-based behavioral prevention intervention	Structured follow-up or service-use pathway	Follow-up after recent assault; Alcohol/marijuana use and related symptoms	Brief secondary prevention after assault
Rajan et al. (2022) [34]	Sweden	Sexual violence	Survivors of single-incident sexual assault trauma; Post-assault psychological treatment	Single-session PTSD intervention	Structured follow-up or service-use pathway	Short-term follow-up; PTSD symptoms	Ultra-brief early trauma intervention
Littleton et al. (2016) [35]	USA	Sexual violence	Survivors with rape-related PTSD; Online therapist-facilitated program	Digital trauma-focused therapy	Remote contact, digital support, or telehealth-enabled continuity	Post-program follow-up; PTSD and anxiety symptoms	Digital post-rape psychotherapy
Hicks et al. (2017) [36]	USA	Sexual violence	Sexual assault patients after examination; Post-exam follow-up program	SMS follow-up / engagement support	Structured follow-up or service-use pathway	Immediate post-examination period; Engagement, feasibility, follow-up contact	Low-cost post-exam continuity tool
Healey et al. (2023) [37]	Australia	Sexual violence	Sexual assault victims referred for medical review; Medical follow-up after assault	Scheduled medical follow-up pathway	Structured follow-up or service-use pathway	Attendance at follow-up visit; Follow-up attendance predictors	Retention in post-assault clinical care
Engleton et al. (2022) [38]	USA	Sexual violence	Sexual assault survivors engaging with advocacy; Advocacy services	Advocacy-based support	Advocacy/case-management linkage and service navigation	Pandemic-period service engagement; Service engagement and barriers	Role of advocacy in continuity of care
Dworkin et al. (2023) [39]	USA	Sexual violence	Recent sexual assault survivors; mHealth early intervention	Digital/mHealth early intervention	Remote contact, digital support, or telehealth-enabled continuity	Early follow-up; Feasibility, acceptability, PTSD and alcohol-related outcomes	Scalable digital post-assault support
Mercier et al. (2024) [40]	Canada	Sexual violence	Survivors of sexual assault and IPV; Telemedicine / virtual care	Telehealth follow-up model	Remote contact, digital support, or telehealth-enabled continuity	Post-acute virtual care pathway; Acceptability, barriers, facilitators	Tele-follow-up for geographically dispersed systems
Johnson et al. (2020) [41]	USA	IPV/domestic	Shelter residents with IPV-related PTSD; Shelter	Shelter-based psychotherapy	Scheduled therapeutic sessions with post-treatment assessment	Post-treatment follow-up; PTSD and mental health symptoms	Embedding therapy inside shelter pathway
Santos et al. (2017) [42]	Portugal	IPV/domestic	Female IPV victims; Group program	Group psychosocial intervention	Scheduled therapeutic sessions with post-treatment assessment	Post-program evaluation; Psychological / empowerment outcomes	Structured group follow-up pathway
Naismith et al. (2021)	Colombia	IPV/domestic	Female survivors of IPV/GBV;	Compassion-based group	Scheduled therapeutic sessions with	Pilot follow-up; Mental health / self-	Low-resource group

Study	Country	Violence type	Population / setting	Post-acute component	Continuity mechanism / workforce	Follow-up / outcomes	Synthesis relevance
[43]			Group therapy	therapy	post-treatment assessment	compassion outcomes	psychological model
Li et al. (2024) [44]	USA	IPV/domestic	Chinese immigrant women experiencing IPV; Community-based support/therapy	Self-compassion / empowerment intervention	Scheduled therapeutic sessions with post-treatment assessment	Pilot follow-up; Health, empowerment, mental health	Culturally tailored intervention for migrant survivors
Cheung et al. (2019) [45]	Hong Kong	IPV/domestic	Chinese women survivors of IPV; Community intervention	Mind-body / Qigong intervention	Scheduled therapeutic sessions with post-treatment assessment	Trial follow-up; Mental health and biologic stress markers	Complementary culturally adapted recovery model
Foschiera et al. (2023) [46]	Brazil	IPV/domestic	Women with history of IPV; Psychotherapy service	Psychotherapy protocol	Scheduled therapeutic sessions with post-treatment assessment	Follow-up study; Psychological outcomes	Longer-term psychotherapy evidence
Andersson et al. (2021) [47]	Sweden	IPV/domestic	Survivors of IPV; Internet-based treatment	Internet-delivered CBT	Remote contact, digital support, or telehealth-enabled continuity	Pilot follow-up; Mental health and feasibility	Digital CBT pathway
Sabri et al. (2024) [48]	USA	IPV/domestic	Abused women at high firearm risk; Digital safety support	Digital safety and support intervention	Remote contact, digital support, or telehealth-enabled continuity	Preliminary efficacy follow-up; Feasibility, acceptability, safety-related outcomes	Digital safety-planning extension
Sullivan et al. (2022) [49]	USA	IPV/domestic	Domestic violence survivors; Housing + advocacy services	Housing First + advocacy	Longitudinal housing support and stabilisation contacts	6 months; Safety and housing stability	Early effects of social stabilization pathway
Sullivan et al. (2023) [50]	USA	IPV/domestic	Domestic violence survivors; Housing + advocacy services	Housing First + advocacy	Longitudinal housing support and stabilisation contacts	12 months; Safety and housing stability	Mid-term trajectory of housing-based model
Nnawulezi et al. (2025) [51]	USA	IPV/domestic	Domestic violence survivors; Housing / trauma-informed services	Trauma-informed practices + housing intervention	Longitudinal housing support and stabilisation contacts	24 months; Safety, housing stability, mental health	Integration of trauma-informed care with social support
Rodgers et al. (2016) [52]	USA	IPV/domestic	Women affected by IPV; Urban community outreach	Community health worker outreach	Structured follow-up or service-use pathway	Program feasibility; Engagement, feasibility, service linkage	Community navigation and follow-up support
Trevillion et al. (2014) [53]	United Kingdom	IPV/domestic	Women accessing IPV advocacy; Advocacy services	Advocacy for recovery	Advocacy/case-management linkage and service navigation	Observational follow-up; Recovery / mental health indicators	Advocacy as continuity mechanism
Brignone et al. (2022) [54]	USA	IPV/domestic	Patients screening positive for IPV; Emergency department to advocacy link	Digital warm handoff to advocacy	Advocacy/case-management linkage and service navigation	Post-ED linkage; Access to advocacy services	Bridge from acute care to downstream services
Halliwell et al. (2019) [55]	United Kingdom	IPV/domestic	Domestic violence and abuse survivors in hospital pathway; Hospital-based advocacy	Hospital-based advocacy intervention	Advocacy/case-management linkage and service navigation	Service evaluation; Healthcare use / referral / support outcomes	Hospital-to-community coordination model
Dheensa et al. (2020) [56]	United Kingdom	IPV/domestic	Service users and staff in DVA pathway; Hospital-based advocacy	Hospital-based advocacy intervention	Advocacy/case-management linkage and service navigation	Implementation evaluation; Acceptability, implementation, barriers	Operational lessons for integrated hospital models
Berry et al. (2024) [57]	USA	IPV/domestic	Survivors served by IPV advocacy organization; Advocacy organization with co-located mental	Co-located specialized mental health services	Advocacy/case-management linkage and service navigation	Program implementation; Service integration / uptake	Physical integration of mental health into advocacy setting

Study	Country	Violence type	Population / setting	Post-acute component	Continuity mechanism / workforce	Follow-up / outcomes	Synthesis relevance
			health care				
Hackenberg et al. (2021) [58]	Finland	IPV/domestic	Victims of severe IPV; Primary care emergency rooms	Advocacy referral gap analysis	Advocacy/case-management linkage and service navigation	Prospective observation; Receipt/non-receipt of advocacy intervention	Identifies missed continuity opportunities after acute contact
Sorrentino et al. (2021) [59]	USA	IPV/domestic	IPV survivors; Mental health care pathway	Survivor perspectives on mental health care	Structured follow-up or service-use pathway	Not intervention-focused; Perceived barriers/facilitators to mental health care	Useful for interpreting service design and acceptability
van Gelder et al. (2023) [60]	Netherlands	IPV/domestic	Women experiencing IPV/abuse; eHealth / remote support	eHealth intervention	Remote contact, digital support, or telehealth-enabled continuity	RCT follow-up; Safety, empowerment, mental health, usability	Scalable remote support model
Rizo et al. (2016) [61]	USA	IPV/domestic	Court/CPS-involved female IPV survivors; System-involved survivor program	Parenting and safety program (MOVE-related)	Structured programme contact and safety/parenting support	Post-program follow-up; Participant experiences, safety, parenting	Complex wraparound program for high-need survivors

Abbreviations: DFSV, domestic, family, and sexual violence; IPV, intimate partner violence; SANE, sexual assault nurse examiner.

Supplementary File S4. Reports excluded from the core synthesis after full-text assessment

Primary reasons for exclusion are reported for all reports assessed in full text that did not meet criteria for the charted core synthesis.

Table S5. Full-text exclusions and primary reasons.

Theme	Citation	Primary reason for exclusion
Sexual violence	Munro-Kramer ML, Cannon LM, Scheiman L, St Ivany AR, Bailey JM. Accessing Healthcare Services During the COVID-19 Pandemic: The Plight of Sexual Assault Survivors. Journal of Forensic Nursing. 2021;17(2):93-97.	Primarily about volume of medical forensic examinations/access during COVID; not a post-acute pathway study.
Sexual violence	Adams M, Fitzgerald S, Holbrook D. Connecting Hispanic Women in Baltimore to the Mercy Medical Center Sexual Assault Forensic Examiners/Forensic Nurse Examiners Program: A Preliminary Assessment of Service Utilization and Community Awareness. Journal of Forensic Nursing. 2016.	Focus on underutilization/access to forensic exam program, not post-acute care.
Sexual violence	Campbell R, Greeson MR, Fehler-Cabral G. With Care and Compassion: Adolescent Sexual Assault Victims' Experiences in Sexual Assault Nurse Examiner Programs. Journal of Forensic Nursing. 2013.	Acute SANE encounter rather than post-acute follow-up.
Sexual violence	Campbell R, Markowitz J, Fedewa T, Shareef S, Fenton D, Southard K. Improving Access to Postassault Healthcare for College Students: Creating a Campus-Based Sexual Assault Nurse Examiner Program. Journal of Forensic Nursing. 2023;19(1):50-59.	Focus on access point/SANE programme without a sufficiently identifiable post-acute continuity component.
Sexual violence	Vandenberghe A, Hendriks B, Peeters L, Roelens K, Keygnaert I. Establishing Sexual Assault Care Centres in Belgium: health	Pre-implementation/provider survey; not concrete

	professionals' role in the patient-centred care for victims of sexual violence. BMC Health Services Research. 2018;18:807.	post-acute service outcomes.
Sexual violence	Serenska A, Sarnquist CC, Darmstadt GL. Variation in rates of sexual assault crisis counsellor usage during forensic examination in California: An observational study. BMJ Open. 2023;13:e072635.	Centered on forensic exam accompaniment during acute encounter.
Sexual violence	Driver M. Enacting an 'empowerment approach' after sexual assault: The views and beliefs of forensic nurse examiners and crisis support workers. Journal of Forensic Nursing. 2024;20(2):E11-E19.	Professional views; limited direct evidence on post-acute pathways.
Sexual violence	Bucossi M, et al. Nonoccupational post-exposure prophylaxis offer, acceptance, and adherence in sexual assault victims. Academic Emergency Medicine. 2017. Conference abstract.	Acute prophylaxis; abstract only.
Sexual violence	Silva de Sousa L, et al. The COVID-19 pandemic and outpatient follow-up of victims of sexual violence in a public health service in Northeast Brazil. Journal of Sexual Medicine. 2023. Conference abstract.	Potentially relevant but abstract only.
Sexual violence	Dworkin ER, et al. Supporter-focused early intervention for recent sexual assault survivors: Study protocol for a pilot randomized clinical trial. Contemporary Clinical Trials. 2022;119:106848.	Protocol only, no outcomes.
Sexual violence	Mercier O, et al. Qualitative analysis of telemedicine and virtual healthcare for sexual assault and intimate partner violence survivors. Canadian Journal of Emergency Medicine. 2023.	Conference abstract duplicate of full 2024 qualitative paper.
IPV/domestic	Gilbert L, Shaw SA, Goddard-Eckrich D, et al. Project WINGS (Women Initiating New Goals of Safety): a randomised controlled trial of a screening, brief intervention and referral to treatment (SBIRT) service to identify and address intimate partner violence victimisation among substance-using women receiving community supervision. Criminal Behaviour and Mental Health. 2015.	Primarily screening/brief intervention/referral model; closer to case identification than post-acute continuity pathway.
IPV/domestic	Gilbert L, Jiwatram-Negron T, Nikitin D, et al. Feasibility and preliminary effects of a screening, brief intervention and referral to treatment model to address gender-based violence among women who use drugs in Kyrgyzstan: Project WINGS. Drug and Alcohol Review. 2017;36(1):125-133.	As above; broad screening/referral model rather than post-acute follow-up care.
IPV/domestic	Tiwari A, Cheung DST, Hui V. Improving mental health outcomes of Chinese women survivors of intimate partner violence through advocacy interventions. Global Mental Health. 2018.	Commentary, not empirical primary study.
IPV/domestic	Messing JT, Campbell J, Webster DW, Brown S, Patchell B, Wilson JS. The Oklahoma Lethality Assessment Study: A Quasi-Experimental Evaluation of the Lethality Assessment Program. Social Service Review. 2015.	Police-linked lethality assessment/referral; more crisis triage than post-acute care.
IPV/domestic	Sigurvinsdottir R. The effects of a Police second response program on domestic violence survivors. Dissertation. 2018.	Police second-response focus, outside healthcare/social care post-acute pathway core.
IPV/domestic	Sudderth LK. Bringing in 'the ones who know them': Informal community and safety planning for victims of intimate partner violence in New Zealand. Violence Against Women. 2017;23(2):222-242.	Important conceptually but less clearly an organized intervention/service study.
IPV/domestic	Sun KJ, Zhu KY, Moon TJ, Breslin MA, Ho VP, Vallier HA. Recovery Services for Interpersonal Violence Victims on Healthcare	Mixed interpersonal violence, not sufficiently specific

	Use at a Trauma Center. Journal of Surgical Research. 2024.	to IPV/sexual violence survivors.
IPV/domestic	Briot B, Habarugira FR, Staring F, Zinnen V. Decentralization of gender-based violence case management services and capacity building in Rwanda: An alternative to improve survivors' accessibility and care? 2023.	Conference abstract only.
IPV/domestic	Vogt C, et al. From needs assessment to usability testing: evaluating the AinoAidTM chatbot for domestic violence support. BMC Women's Health. 2025;26:31.	Technology support/usability study, broad DV support rather than post-acute care pathway.
IPV/domestic	Dasgupta S, Melvin E. Technology-Based Intimate Partner Violence Intervention Services for Generation Z Victims of Violence. Social Sciences. 2024;13(3):169.	Review/discussion piece, not primary intervention study.
IPV/domestic	Uysal J, Boyce SC, Undie CC, Liambila W, Wendoh S, Pearson E, Johns NE, Silverman JG. Effects of a clinic-based reproductive empowerment intervention on proximal outcomes... Sexual and Reproductive Health Matters. 2023.	Reproductive empowerment is adjacent but not clearly a post-acute violence follow-up pathway.
Sexual violence	Yang D, Sherak R, Schenck C, Gawel M, Cordone A, Sun W, Rhodes D, Dodington J, Sangal R. Improved Care for Survivors of Sexual Assault With an Electronic Health Record-Integrated Clinical Pathway.	Conference abstract only; insufficient full-text data for core synthesis.
Sexual violence	Cannon LM, Munro-Kramer ML, Scheiman L, et al. Patient satisfaction with SANE services and post-assault resource utilization.	Service satisfaction/resource-utilization paper; informative for background but not a discrete post-acute pathway for the core synthesis.
Sexual violence	Hoffman EE, Zinter KE, Greeson MR, et al. Medical advocates’ care for sexual assault survivors.	Advocacy-care context; useful as background but not sufficiently centred on an identifiable post-acute continuity model.
IPV/domestic	Rhodes KV, Rodgers M, Sommers M, et al. Brief motivational intervention for IPV and heavy drinking in the emergency department.	Emergency-department brief intervention with screening/brief counselling emphasis; closer to acute identification than to a post-acute pathway.
IPV/domestic	Weiss M, Benavides MO, Fitelson E, Monk C. Domestic Violence Initiative providing psychiatric care in a nontraditional setting.	Psychiatric-care initiative in an adjacent service setting; insufficiently described as a structured post-acute continuity pathway.
IPV/domestic	Li Y, Dong FH, Bullock LFC, Bloom T. Help-seeking experiences of Chinese immigrant IPV survivors in the U.S.	Help-seeking and access-barrier study; relevant to interpretation but not an intervention or pathway study for core charting.
Sexual violence	Brooker C, Durmaz E. Mental health, sexual violence and the work of Sexual Assault Referral centres (SARCs) in England.	Service-level evidence on mental-health assessment and referral; interpretive background rather than a chartable post-acute intervention study.
Mixed	Macy RJ, Martin SL, Ogbonnaya IN, Rizo CF. What Do Domestic Violence and Sexual Assault Service Providers Need to Know About Survivors to Deliver Services?	Provider assessment and service-planning study; relevant to tailoring but not a discrete post-acute continuity pathway.

Mixed	Storer HL, Nyerges EX. The Rapid Uptake of Digital Technologies at Domestic Violence and Sexual Assault Organizations During the COVID-19 Pandemic.	Organisational adaptation to virtual services; relevant to context but not a specific evaluated post-acute pathway.
IPV/domestic	Muftić LR, Hoppe S, Grubb JA. The use of help seeking and coping strategies among Bosnian women in domestic violence shelters.	Shelter-based help-seeking/coping study; informative for barriers and retention but not an organised post-acute intervention.
Mixed	Iraola E, Menard J-P, Baranne M-L, Cudonnec J, Buresi I, Chariot P. Low uptake of gynecological consultation following domestic or sexual violence: A case-control study during pregnancy follow-up.	Follow-up utilization study; important for service-underuse interpretation but not a structured continuity model.
Mixed	Pallansch J, Milam C, Ham K, Morgan P, Manning J, Salzman J, Kopec K, Lewis M. Intimate Partner Violence, Sexual Assault, and Child Abuse Resource Utilization During COVID-19.	Resource-utilization study relevant to access disruption, but not a discrete post-acute care pathway.
Sexual violence	Bach MH, Krogh SNS, Hansen M. "That kind of information is crucial to get across": co-developing a sexual assault support website with survivors and support providers.	Co-designed support-resource study; relevant to survivor-informed digital support but not an evaluated post-acute pathway.
IPV/domestic	Hooker L, Taft A. Using theory to design, implement and evaluate sustained nurse domestic violence screening and supportive care.	Supportive care and referral implementation paper; partly screening-led rather than a chartable post-acute pathway.
IPV/domestic	Meyer JP, Lazarus E, Phillips K, Watts ZT, Duroseau B, Carlson C, Price CR, Kershaw T, Willie TC. A PrEP decision aid for women survivors of intimate partner violence: Task-shifting implementation to domestic violence service settings.	Implementation study in domestic violence agencies; adjacent preventive-health focus rather than a post-acute violence-care pathway.

Note. Table S5 is a methodological audit of full-text exclusions. Bracketed reference numbers are shown only where the excluded report is already included in the main manuscript reference list for contextual reasons. Unnumbered entries are retained as bibliographic exclusion records only; they are not part of the main manuscript reference list, the charted core synthesis, or numerical citation callouts.
